# Afadin cooperates with Claudin-2 to promote breast cancer metastasis

**DOI:** 10.1101/gad.319194.118

**Published:** 2019-02-01

**Authors:** Sébastien Tabariès, Alexander McNulty, Véronique Ouellet, Matthew G. Annis, Mireille Dessureault, Maude Vinette, Yasmina Hachem, Brennan Lavoie, Atilla Omeroglu, Hans-Georg Simon, Logan A. Walsh, Siker Kimbung, Ingrid Hedenfalk, Peter M. Siegel

**Affiliations:** 1 Goodman Cancer Research Centre, McGill University, Montréal, Québec H3A 1A3, Canada;; 2 Department of Medicine, McGill University, Montréal, Québec H3A 1A3, Canada;; 3 Department of Biochemistry, McGill University, Montréal, Québec H3A 1A3, Canada;; 4 Institut du Cancer de Montréal, Centre de Recherche du Centre Hospitalier de l'Université de Montréal, Montréal, Québec H2X 0A9, Canada;; 5 Department of Pathology, McGill University Health Centre, Montréal, Québec H4A 3J1, Canada;; 6 Department of Pediatrics, Feinberg School of Medicine, Northwestern University, Chicago, Illinois 60614, USA;; 7 Stanley Manne Children's Research Institute, Chicago, Illinois 60614, USA;; 8 Department of Human Genetics, McGill University, Montréal, Québec H3A 1A3, Canada;; 9 Division of Oncology, Department of Clinical Sciences, Lund University, Lund SE 221 00, Sweden

**Keywords:** breast cancer, liver metastasis, lung metastasis, Claudin-2, Afadin

## Abstract

Claudin-2 promotes breast cancer liver metastasis by enabling seeding and early cancer cell survival. We now demonstrate that the PDZ-binding motif of Claudin-2 is necessary for anchorage-independent growth of cancer cells and is required for liver metastasis. Several PDZ domain-containing proteins were identified that interact with the PDZ-binding motif of Claudin-2 in liver metastatic breast cancer cells, including Afadin, Arhgap21, Pdlim2, Pdlim7, Rims2, Scrib, and ZO-1. We specifically examined the role of Afadin as a potential Claudin-2-interacting partner that promotes breast cancer liver metastasis. Afadin associates with Claudin-2, an interaction that requires the PDZ-binding motif of Claudin-2. Loss of Afadin also impairs the ability of breast cancer cells to form colonies in soft agar and metastasize to the lungs or liver. Immunohistochemical analysis of Claudin-2 and/or Afadin expression in 206 metastatic breast cancer tumors revealed that high levels of both Claudin-2 and Afadin in primary tumors were associated with poor disease-specific survival, relapse-free survival, lung-specific relapse, and liver-specific relapse. Our findings indicate that signaling downstream from a Claudin-2/Afadin complex enables the efficient formation of breast cancer metastases. Moreover, combining Claudin-2 and Afadin as prognostic markers better predicts the potential of breast cancer to metastasize to soft tissues.

## Abstract (precis)

Tabariès et al. show that signaling downstream from a Claudin-2/Afadin complex enables the efficient formation of breast cancer metastases.

Claudins are key components of tight junctions and have emerged as important regulators of the metastatic cascade (Agarwal et al. 2005; Martínez-Estrada et al. 2006; Martin et al. 2011; Escudero-Esparza et al. 2012; Tabariès and Siegel 2017). Claudins are tetraspan transmembrane proteins consisting of two extracellular loops, an intracellular N terminus, and a cytoplasmic C-terminal tail (Tabariès and Siegel 2017). Claudin proteins possess a PDZ-binding motif within the C terminus that mediates binding to tight junction scaffolding proteins, such as MUPP1 and members of the MAGUK protein family (Tabariès and Siegel 2017). The PDZ-binding motif of Claudin proteins is comprised of the three C-terminal amino acids, within which tyrosine and valine residues are invariant at position 0 and −1, respectively, and the −2 position is variable (Krause et al. 2008). The importance of PDZ domain/PDZ-binding motif-mediated interactions in cancer progression is starting to be recognized. For example, the PDZ-binding motif of Claudin-1 is required to enable anoikis resistance in colon cancer cells by recruiting Src into a complex with ZO-1 (Singh et al. 2012).

Claudin-2 is emerging as a promoter of cancer progression and metastasis. Claudin-2 expression is increased in colorectal and gastric cancers, both of which are highly metastatic to the liver (Aung et al. 2006; Kinugasa et al. 2007; Dhawan et al. 2011; Jung et al. 2011). Furthermore, Claudin-2 expression increases the tumorigenicity of colorectal cancer cells by enabling anchorage-independent growth (Buchert et al. 2010; Dhawan et al. 2011). In breast cancer, Claudin-2 expression is detected in 52% of breast carcinomas (Soini 2004). Decreased Claudin-2 expression is observed in breast cancers of increasing stage and grade and is associated with lymph node metastasis (Soini 2004; Kim et al. 2008; Szasz et al. 2011). However, Claudin-2 is selectively enriched in breast cancer liver metastases, and high Claudin-2 expression in primary breast tumors is associated with liver-specific metastatic recurrence (Tabariès et al. 2011; Kimbung et al. 2014). Claudin-2 promotes breast cancer liver metastasis by enhancing breast cancer cell interactions with constituents of the extracellular matrix and with hepatocytes (Tabariès et al. 2011, 2012). However, the involvement of the PDZ-binding motif of Claudin-2 in breast cancer metastasis to the liver is not currently known.

In this study, we demonstrate that Claudin-2 expression in breast cancer cells is required for efficient anchorage-independent growth. The PDZ-binding motif of Claudin-2 is required to enable the in vitro growth of breast cancer cells in soft agar and promote the formation of breast cancer liver metastases in vivo. We identified Afadin as a potential binding partner that interacts via the PDZ-binding motif within Claudin-2. Loss of Afadin phenocopies Claudin-2 loss, resulting in impaired growth of breast cancer cells in soft agar and diminished lung or liver metastatic capacity. Two isoforms of Afadin exist, which include a short and long form of the protein. We show that expression of either the long or short Afadin isoforms partially rescued the ability of breast cancer cells to form liver metastases. Finally, we explored the potential of Claudin-2 and/or Afadin as biomarkers to predict metastasis in primary breast cancer. Our results demonstrate a functional requirement for Afadin in promoting breast cancer metastasis to the lungs or liver through a mechanism that may involve complex formation with Claudin-2.

## Results

### Claudin-2 is required for increased anchorage-independent growth of liver metastatic breast cancer cells

**Figure 1.**
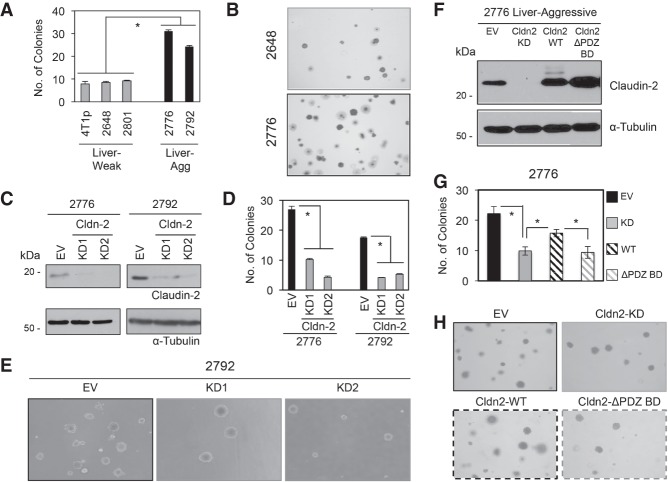
The PDZ-binding motif of Claudin-2 is required for efficient anchorage-independent growth of aggressively liver metastatic 4T1-derived breast cancer cells. (*A*) Growth of liver-weak (4T1p, 2648, and 2801) and liver-aggressive (2776 and 2792) breast cancer cells in soft agar. (*) *P* < 0.0001. (*B*) Representative pictures of colony formation by liver-weak (2648) and liver-aggressive (2776) breast cancer cells are shown. (*C*) Immunoblot analysis of Claudin-2 expression in liver-aggressive cells (2776 and 2792) infected with independent *Claudin-2* shRNA expression vectors (knockdown [KD1 and KD2]) or harboring an empty vector (EV). As a loading control, total cell lysates were blotted for α-Tubulin. (*D*) Soft agar assays using these breast cancer cells were performed, and colony-forming ability was quantified. (*) *P* < 0.0001. (*E*) Representative images of colony formation by liver-aggressive cells with reduced Claudin-2 levels (knockdown [KD1 and KD2]) or control cells (empty vector [EV]) are shown. (*F*) Immunoblot analysis of Claudin-2 expression in the indicated cell lines. Wild-type Claudin-2 and the Claudin-2 mutant lacking the PDZ-binding motif (Cldn2 ΔPDZ BD) were expressed in 2776 liver-aggressive breast cancer cells lacking endogenous Claudin-2. Pooled populations of individual clones (*n* = 3) expressing either wild-type Claudin-2 or the Claudin-2 ΔPDZ BD mutant are shown. Immunoblot analysis of α-Tubulin served as a loading control. (*G*) Colony formation of the indicated cells lines in soft agar was analyzed. (*) *P* < 0.000004. (*H*) Representative images of colony growth formation in soft agar are presented for each cell population.

Claudin-2 promotes anchorage-independent growth of colorectal cancer cells in soft agar (Buchert et al. 2010; Dhawan et al. 2011). We previously isolated weakly and aggressively liver metastatic 4T1 breast cancer cell populations and discovered that Claudin-2 was elevated in the latter (Tabariès et al. 2011). To investigate whether Claudin-2 conferred anchorage-independent growth to liver metastatic 4T1 breast cancer cells, we assessed the ability of weakly and aggressively liver metastatic cell lines to grow in soft agar. Aggressively liver metastatic cells showed a 3.24-fold increase in colony-forming ability in soft agar compared with the weakly liver metastatic cells (Fig. 1A,B).

To determine whether Claudin-2 was responsible for the observed increase in anchorage-independent growth, we stably diminished endogenous Claudin-2 levels in two aggressively liver metastatic 4T1 breast cancer cell populations using two independent *Claudin-2* shRNA expression vectors (Fig. 1C; Tabariès et al. 2011). Aggressively liver metastatic cell populations with diminished Claudin-2 levels demonstrated a 3.71-fold to 3.74-fold reduction in colony-forming ability in soft agar when compared with their empty vector controls (Fig. 1D,E). These results indicate that Claudin-2 enhances the ability of aggressively liver metastatic breast cancer cells to form colonies in soft agar.

### The PDZ-binding motif of Claudin-2 is required for enhanced colony formation of breast cancer cells in soft agar

We next determined the functional contribution of the PDZ-binding motif within Claudin-2 in promoting the ability of aggressively liver metastatic cells to grow in soft agar. We first engineered weakly liver metastatic breast cancer cells to harbor an empty vector or overexpress either a wild-type or a mutant form of Claudin-2. The mutant form of Claudin-2 lacks the three C-terminal amino acids that constitute the PDZ-binding domain (Cldn2 ΔPDZ BD) (Supplemental Fig. S1A; Van Itallie et al. 2004). As expected, weakly liver metastatic breast cancer cells overexpressing Claudin-2 exhibited a 3.26-fold to 4.20-fold increase in anchorage-independent colony formation compared with their respective vector controls (Supplemental Fig. S1B–D). Weakly liver metastatic cells overexpressing Cldn2 ΔPDZ BD failed to efficiently form colonies in soft agar (Supplemental Fig. S1B–D). These results suggest that the PDZ-binding motif is required for Claudin-2-mediated anchorage-independent growth of weakly liver metastatic breast cancer cells.

Using liver metastatic 4T1 subpopulations with stably diminished Claudin-2 expression (Fig. 1C; Tabariès et al. 2012), we engineered these cells to express either wild-type Claudin-2 (Cldn2 wild type) or Cldn2 ΔPDZ BD (Van Itallie et al. 2004). Immunoblot analyses were performed to identify individual clones that expressed either the wild-type or mutant form of Claudin-2. To reduce the possibility of clonal variation interfering with our results, we created pooled populations of individual clones (*n* = 3 per pool) expressing Cldn-2 wild type or Cldn2 ΔPDZ BD (Fig. 1F). Consistent with our previous results (Fig. 1C–E), knockdown of Claudin-2 resulted in 2.33-fold fewer colonies that formed in soft agar compared with empty vector controls (Fig. 1G,H). Importantly, while expression of wild-type Claudin-2 was able to significantly rescue colony formation relative to breast cancer cells with knockdown of endogenous Claudin-2, the pooled population of liver metastatic breast cancer cells expressing the Claudin-2 ΔPDZ BD mutant failed to efficiently form colonies in soft agar (Fig. 1G,H). Thus, the PDZ-binding motif in Claudin-2 is required for anchorage-independent growth of aggressively liver metastatic breast cancer cells.

### The PDZ-binding motif is dispensable for Claudin-2-mediated adhesion to hepatocytes and extracellular matrix components

Our previous studies revealed that Claudin-2 enhances breast cancer cell adhesion to hepatocytes through Claudin-2-dependent homotypic interactions (Tabariès et al. 2012). As reported (Tabariès et al. 2012), reduced Claudin-2 expression resulted in a 2.3-fold decrease in cancer cell adhesion to hepatocytes (Supplemental Fig. S2A,B). Importantly, expression of either wild-type or a ΔPDZ BD mutant form of Claudin-2 fully restores the ability of these breast cancer cells to adhere to primary hepatocytes (Supplemental Fig. S2A,B). These data indicate that the PDZ-binding motif is not required for Claudin-2-mediated adhesion to primary hepatocytes.

We also demonstrated that Claudin-2 increases the formation of α_2_β_1_ and α_5_β_1_ integrin complexes at the cell membrane, which enhances breast cancer adhesion to collagen IV and fibronectin (Tabariès et al. 2011). In agreement with our previous results (Tabariès et al. 2011), diminished Claudin-2 expression is accompanied by a reduction in the ability of the aggressively liver metastatic cells to adhere to both fibronectin and collagen IV (Supplemental Fig. S2C–F). However, expression of either wild-type Claudin-2 or the ΔPDZ BD mutant can restore adhesion to fibronectin (Supplemental Fig. S2C,D) and partially restore adhesion to collagen IV (Supplemental Fig. S2E,F). Thus, the Claudin-2 PDZ-binding motif is dispensable for adhesion to fibronectin or collagen IV.

### The PDZ-binding motif of Claudin-2 is required for liver metastasis

**Figure 2.**
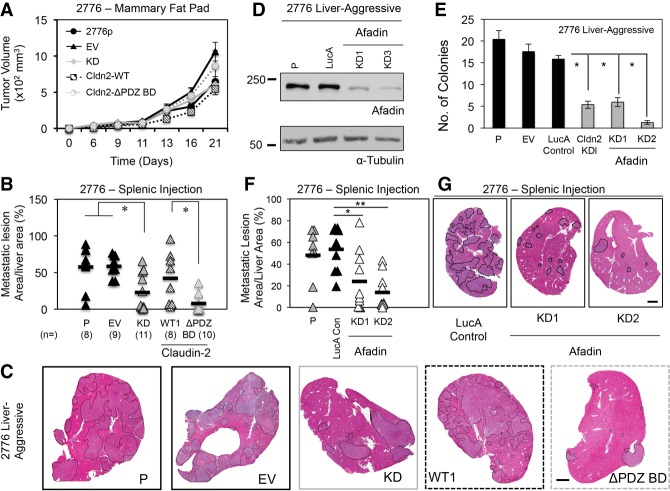
The Claudin-2 PDZ-binding motif and Afadin are required for efficient liver metastasis. (*A*) Tumor growth in the mammary fat pad was measured for the indicated liver-aggressive (2776) cell populations: parental (2776p), empty vector (EV), *Claudin-2* shRNA expression vector (knockdown), a pooled population expressing wild-type Claudin-2, and a pooled population expressing a Claudin-2 mutant lacking three C-terminal amino acids (ΔPDZ BD). (*B*) Liver metastatic burden (tumor area per tissue area) was analyzed after splenic injection of the indicated cell lines. (*) *P* ≤ 0.005. The number of mice analyzed in each cohort is shown in parentheses. (*C*) Representative images of the cardiac liver lobe are shown. Metastatic lesions are indicated by dotted lines. Bar , 2 mm. (*D*) Afadin knockdown in liver-aggressive cells was assessed by immunoblot analysis, and α-Tubulin served as a loading control. (*E*) Colony formation in soft agar was assessed for the indicated cell populations. (*) *P* < 0.00001. (*F*) Liver metastatic burden (tumor area per tissue area) was analyzed following splenic injection for the indicated cell lines. (*) *P* < 0.008; (**) *P* < 0.0002. (*G*) Representative images of the cardiac liver lobe for each cell population are shown. Metastatic lesions are outlined by dotted lines. Bar, 2 mm.

Next, we examined the importance of the PDZ-binding motif for Claudin-2-mediated liver metastasis using the pooled liver-aggressive populations expressing either wild-type Claudin-2 or the ΔPDZ BD mutant. No significant change in the growth of mammary tumors was observed between parental (2776p), empty vector, knockdown, pooled wild-type (Cldn2 wild-type), or pooled mutant 4T1 populations (Cldn2 ΔPDZ BD) (Fig. 2A). Reducing Claudin-2 expression resulted in a 2.57-fold decrease in liver metastatic burden compared with parental and empty vector controls following splenic injections (Fig. 2B,C). The pooled population expressing wild-type Claudin-2 produced a similar metastatic burden relative to the parental and empty vector control populations, whereas the Cldn2 ΔPDZ BD-expressing pooled population did not (Fig. 2B,C). The ability of wild-type Claudin-2-expressing breast cancer cells to form liver metastases was increased 5.56-fold compared with the Claudin-2 ΔPDZ BD-expressing population (Fig. 2B,C). These data demonstrate that the PDZ-binding motif within Claudin-2 is required for efficient breast cancer liver metastasis.

### Identification of PDZ domain-containing proteins that interact with wild-type Claudin-2 but not the PDZ BD mutant

To investigate potential binding partners that interact via the PDZ-binding motif of Claudin-2, we generated hemagglutinin (HA)-tagged versions of both wild-type and the Claudin-2 ΔPDZ BD mutant and expressed them in liver-aggressive 4T1 breast cancer cells (2776) (Supplemental Fig. S3A). We performed anti-HA immunoprecipitations from these cells followed by silver staining to identify potential binding partners that interact via the Claudin-2 PDZ-binding motif (Supplemental Fig. S3B). Mass spectrometry analysis performed on excised gel pieces identified >100 potential candidate proteins that were immunoprecipitated with wild-type Claudin-2 that were not detected in immunoprecipitates of the Claudin-2 ΔPDZ BD mutant. To interrogate the potential functional relevance of these proteins to liver metastasis, we initially restricted our focus to the seven candidates that possess a PDZ domain (Table 1). Identification of ZO-1 validated the immunoprecipitation/mass spectrometry approach, as the PDZ-binding motif of Claudin-2 is known to bind the PDZ domain of ZO-1 in epithelial cells (Itoh et al. 1999; Rodgers et al. 2013). We restricted our analyses to the six remaining PDZ domain-containing proteins that represent potential novel partners for Claudin-2.

**Table 1.**
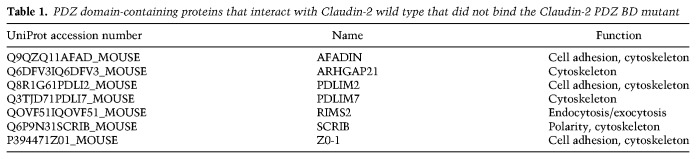
PDZ domain-containing proteins that interact with Claudin-2 wild type that did not bind the Claudin-2 PDZ BD mutant

### Pdlim7 and Afadin are required for efficient breast cancer metastasis to the liver

To assess whether these potential interacting proteins promote Claudin-2 dependent phenotypes, we stably diminished their expression in the 2776 liver-aggressive cells using shRNA-mediated approaches and assessed the impact on anchorage-independent growth. Diminished expression of *Arhgap21* (Supplemental Fig. S4A,B) and *Rims2* (Supplemental Fig. S4B,D) resulted in an up to 1.79-fold and 13.18-fold reduction in soft agar colony formation when compared with controls (empty vector), suggesting that these two proteins could represent interesting candidates for further study. However, the lack of good-quality antibody reagents against Arhgap21 and Rims2 precluded us from investigating them further with respect to Claudin-2-mediated liver metastasis. In contrast, reduction of Pdlim2 expression failed to negatively impact the growth of liver-aggressive breast cancer cells in soft agar, suggesting that this candidate was not important for Claudin-2-mediated anchorage-independent growth (Supplemental Fig. S4E,F).

Liver-aggressive breast cancer cells with diminished Scrib expression (Supplemental Fig. S5A) exhibited a 2.28-fold reduction in soft agar colony formation relative to controls (empty vector) (Supplemental Fig. S5B). However, reduced Scrib expression did not negatively impact the ability of liver-aggressive breast cancer cells to form liver metastases following splenic injection (Supplemental Fig. S5C,D). These data exclude Scrib as an important Claudin-2-interacting partner that contributes to the liver metastatic phenotype.

Stable reduction of Pdlim7 levels (Supplemental Fig. S6A) led to a 6.05-fold reduction in soft agar colony formation when compared with controls (empty vector and LucA) (Supplemental Fig. S6B) and a 1.96-fold to 2.21-fold decrease in liver metastatic burden compared with controls (LucA) (Supplemental Fig. S6C,D). Similarly, diminished Afadin expression (Fig. 2D) resulted in an up to 13.87-fold reduction in soft agar colony-forming ability (Fig. 2E) and a 2.24-fold to 3.85-fold suppression of liver metastasis relative to controls (LucA) (Fig. 2F,G). Together, these data demonstrate that Pdlim7 and Afadin are functionally involved in promoting the in vitro growth of breast cancer cells in soft agar and the formation of liver metastases in vivo. However, due to limited access to high-quality Pdlim7 antibodies, we prioritized our efforts on investigating the interaction between Claudin-2 and Afadin.

### Loss of Afadin in human breast cancer cells results in diminished liver metastasis

**Figure 3.**
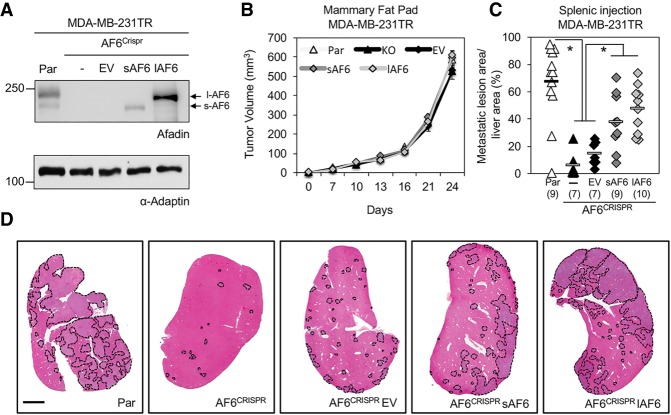
Afadin-deficient human breast cancer cells are impaired in their ability to metastasize to the liver. (*A*) A pooled population (*n* = 3 independent clones) of MDA-MB-231TR cells lacking Afadin (AF6^Crispr^) or harboring either an empty vector (EV), short Afadin (sAF6), or long Afadin (lAF6) were analyzed by immunoblot analysis, with α-Tubulin serving as a loading control. (*B*) Tumor growth in the mammary fat pad was measured for the cell populations described in *A*. (*C*) Liver metastatic burden (tumor area per tissue area) was analyzed following splenic injection of Afadin-proficient and Afadin-deficient MDA-MB-231TR breast cancer cells. (*) *P* < 2 × 10^−3^. (*D*) Representative images of the cardiac liver lobe for each cell population are shown. Metastatic lesions are outlined by dotted lines. Bar, 2 mm.

We next assessed the contribution of Afadin to the formation of breast cancer liver metastases in an independent cell model. An Afadin knockout was generated in MDA-MB-231TR breast cancer cells that harbor a triple-modality reporter (Minn et al. 2005) using CRISPR/Cas9 approaches. Independent clones were first screened by immunoblot to identify those with loss of Afadin expression, and a pool of three clones lacking Afadin was established (AF6^Crispr^) (Fig. 3A). To ensure that potential phenotypes observed in AF6^Crispr^ breast cancer cells were not due to off-target effects, we performed a rescue with two isoforms of Afadin expressed in MDA-MB-231TR cells, which include a short (sAF6) or long (lAF6) isoform of Afadin (Fig. 3A). No significant change in the growth of mammary tumors was observed between parental, AF6^Crispr^, empty vector, sAF6, or lAF6 populations (Fig. 3B). MDA-MB-231TR cells lacking Afadin (AF6^Crispr^ or AF6^Crispr^ empty vector) were severely impaired in their ability to form liver metastases following splenic injection, exhibiting an 11.8-fold reduction in liver metastatic burden when compared with the MDA-MB-231TR parental controls expressing endogenous Afadin (Fig. 3C,D). Pooled populations re-expressing either sAF6 or lAF6 produced a partial rescue of the metastatic burden relative to control populations lacking Afadin, resulting in a 2.6-fold and 3.3-fold increase in metastatic burden, respectively, when compared with AF6^Crispr^ empty vector cells (Fig. 3C,D). Thus, the reduction in liver metastasis observed in mice injected with Afadin-deficient breast cancer cells is similar to that observed in mice injected with Claudin-2 knockdown cells (Fig. 2), and both Afadin isoforms contribute to the formation of breast cancer liver metastases. Together, these data suggest that, like Claudin-2, Afadin functions to promote breast cancer liver metastasis.

### Loss of Afadin or Claudin-2 in human breast cancer cells is also associated with reduced formation of lung metastases

**Figure 4.**
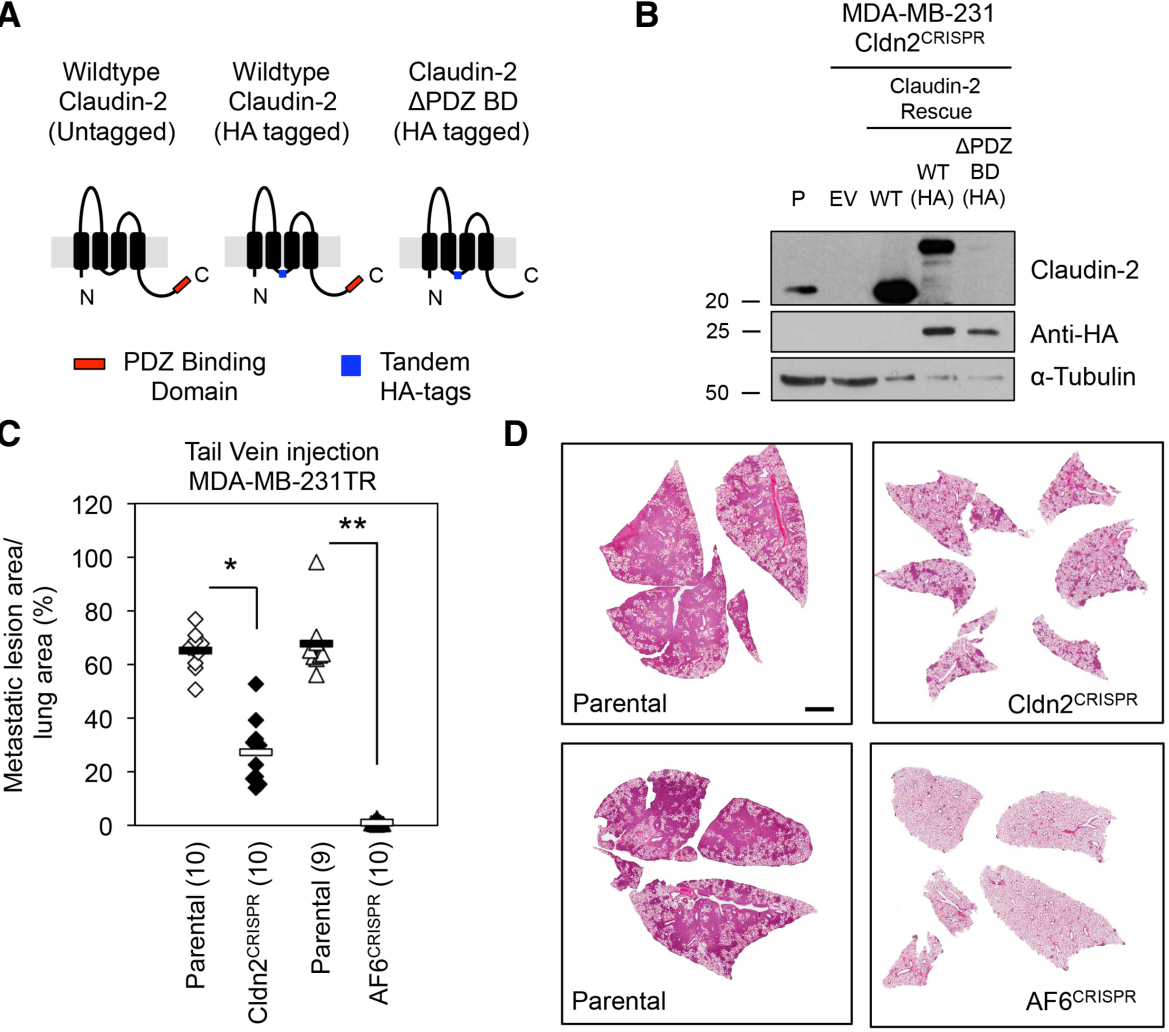
Claudin-2- or Afadin-deficient breast cancer cells are impaired in their ability to metastasize to the lung. (*A*) Schematic of Claudin-2 indicating the presence of the H-influenza HA tag in the cytoplasmic loop of wild-type Claudin-2 and the ΔPDZ BD Claudin-2 mutant. (*B*) Claudin-2 expression in the indicated MDA-MB-231-derived cell populations was analyzed by immunoblotting with anti-Claudin-2 and anti-HA antibodies. α-Tubulin served as a loading control. (*C*) Lung metastatic burden (tumor area per tissue area) was analyzed following tail vein injection of Claudin-2- and Afadin-proficient and Claudin-2- and Afadin-deficient MDA-MB-231 breast cancer cells. (*) *P* < 3 × 10^7^; (**) *P* < 9 × 10^8^. (*D*) Representative images of the lungs for each cell population are shown. Bar, 2 mm.

To determine whether the metastasis-promoting effects of Claudin-2 and Afadin were restricted to the liver, we next assessed their contribution to the formation of breast cancer lung metastases. A knockout of Claudin-2 was generated in MDA-MB-231 breast cancer cells using a CRISPR/Cas9 approach (Tabariès et al. 2011). In the context of endogenous Claudin-2 loss, we engineered HA-tagged versions of both wild-type Claudin-2 and a Claudin-2 ΔPDZ BD mutant (Fig. 4A). Immunoblot analysis confirmed loss of endogenous Claudin-2 and expression of untagged wild-type Claudin-2, HA-tagged wild-type Claudin-2, and the HA-tagged Claudin-2 ΔPDZ BD mutant (Fig. 4B).

Tail vein injections revealed that Claudin-2-deficient cells (Cldn2^Crispr^) exhibited a 2.4-fold reduction in lung metastatic burden when compared with the parental controls that expressed endogenous Claudin-2 (Fig. 4C,D). Interestingly, MDA-MB-231 cells lacking Afadin (AF6^Crispr^) were dramatically impaired in their ability to form lung metastases following tail vein injection, exhibiting a 57-fold reduction in lung metastatic burden when compared with the parental controls expressing endogenous Afadin (Fig. 4C,D). Thus, both Claudin-2 and Afadin not only contribute to the formation of breast cancer liver metastases but also function to promote breast cancer lung metastasis.

### Claudin-2 and Afadin are present in membrane and nuclear fractions of breast cancer cells

**Figure 5.**
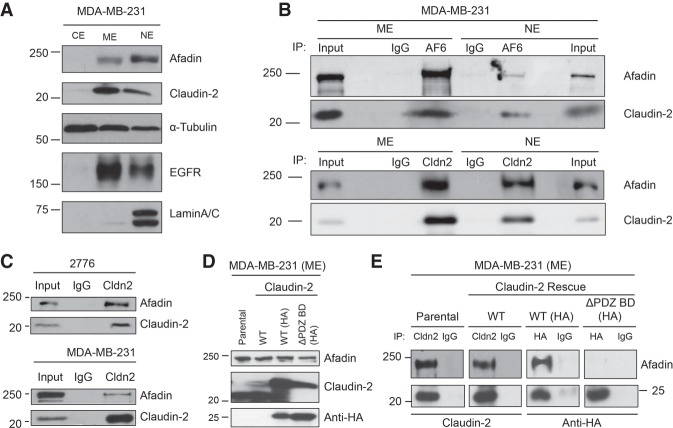
Afadin associates with Claudin-2 via the C-terminal PDZ-binding motif of Claudin-2. (*A*) Immunoblot analysis of Claudin-2 and Afadin expression in subcellular fractions of MDA-MB-231 breast cancer cells. Immunoblots for α-Tubulin served as a control for the cytoplasmic fraction, EGFR was used as a membrane fraction control, and Lamin A/C was used as a nuclear extract control. (*B*) Membrane and nuclear fractions of MDA-MB-231 breast cancer cells were subjected to reciprocal immunoprecipitation analysis using either Claudin-2 or Afadin antibodies. Immunoprecipitates were also generated using IgG isotype control antibodies. (*C*) Coimmunoprecipitation analysis for Claudin-2 and Afadin were conducted using whole-cell lysates from the 2776 liver-aggressive 4T1 breast cancer cell line. (*D*,*E*) The membrane fractions (membrane extract [ME]) from MDA-MB-231 breast cancer cells (*D*) were subjected to coimmunoprecipitation analysis using Claudin-2 or HA antibodies (*E*). Immunoprecipitations were also performed with IgG isotype control antibodies.

Afadin is localized at the membrane as a constituent of adherens junctions and within the nucleus (Mandai et al. 1997; Buchert et al. 2007; VanLeeuwen et al. 2014; Xu et al. 2015). To investigate the interaction between Claudin-2 and Afadin, we first assessed the cytoplasmic and nuclear distribution of both Claudin-2 and Afadin. We extended our analysis to the MDA-MB-231 human triple-negative breast cancer (TNBC) cell line, which expresses high levels of Claudin-2 (Tabariès et al. 2011). Subcellular fractionation of MDA-MB-231 cells was performed to isolate cytoplasmic, membrane, and nuclear extracts, which revealed the presence of Claudin-2 primarily in the membrane fraction (Fig. 5A). The weak Claudin-2 signal detected in the nuclear fraction may reflect contamination from the membrane extract , as suggested by the detection of residual EGFR signal in the nuclear extract samples (Fig. 5A). Conversely, Afadin was localized primarily in the nuclear fraction, with detectable amounts in the membrane extract (Fig. 5A). Reciprocal coimmunoprecipitation experiments from either the membrane or nuclear fractions of MDA-MB-231 cells revealed that Claudin-2 associates with Afadin primarily at the membrane, with a much weaker association detected in the nucleus (Fig. 5B). Reciprocal coimmunoprecipitation analyses of whole-cell lysates from 2776 liver-aggressive cells revealed a clear association between Claudin-2 and Afadin (Fig. 5C).

### The Claudin-2/Afadin association requires the PDZ-binding motif in Claudin-2

Using membrane extracts from MDA-MB-231 cells expressing endogenous Claudin-2 (parental), wild-type Claudin-2, HA-tagged wild-type [wild type (HA)], or HA-tagged Claudin-2 lacking the PDZ-binding motif [ΔPDZ BD(HA)] (Fig. 5D), we performed coimmunoprecipitation experiments to examine the association between Claudin-2 and Afadin. Immunoprecipitation of Claudin-2 revealed an interaction with Afadin in the parental cells and those expressing the untagged or HA-tagged versions of wild-type Claudin-2 (Fig. 5E). Similarly, immunoprecipitation of HA-tagged wild-type Claudin-2 using sepharose beads preconjugated with anti-HA antibodies detected an association between Claudin-2 and Afadin. The interaction between Claudin-2 and Afadin was lost in cells expressing the Claudin-2 ΔPDZ BD(HA) construct (Fig. 5E). Together, these results suggest that Afadin interacts with the PDZ-binding motif within Claudin-2.

### Claudin-2 and Afadin expression is associated with the triple-negative subtype of human breast cancer

Claudin-2 expression in primary breast tumors is prognostic for the development of liver metastases (Kimbung et al. 2014). Using this previously described tumor microarray (TMA) (Kimbung et al. 2014), we performed immunohistochemical staining for Claudin-2 or Afadin (Supplemental Fig. S7A). We first assessed the association of these proteins with histological subtypes of breast cancer, which revealed that both Claudin-2 and Afadin are significantly elevated in the TNBC compared with estrogen receptor (ER^+^) or HER2 subtypes (Supplemental Fig. S7B,C). We next interrogated publicly available RNA sequencing data sets from The Cancer Genome Atlas (TCGA) for associations between *CLDN2* and *AFDN* mRNA expression and breast cancer subtype and outcome. *CLDN2* mRNA expression levels in the TCGA data set were very low across all samples, making it difficult to draw reliable conclusions from the expression data. However, in agreement with the Afadin immunostaining (Supplemental Fig. S7C), *AFDN* mRNA expression is significantly elevated in TNBC (Supplemental Fig. S7D). We partitioned all breast cancer samples into two groups: those with high *AFDN* and those with low *AFDN* expression (Supplemental Fig. S7E). Interestingly, patients with high *AFDN* expression experienced poor overall survival compared with patients with low *AFDN* expression (Supplemental Fig. S7F).

### Claudin-2 and Afadin expression in human metastatic breast cancer predicts liver metastasis

**Figure 6.**
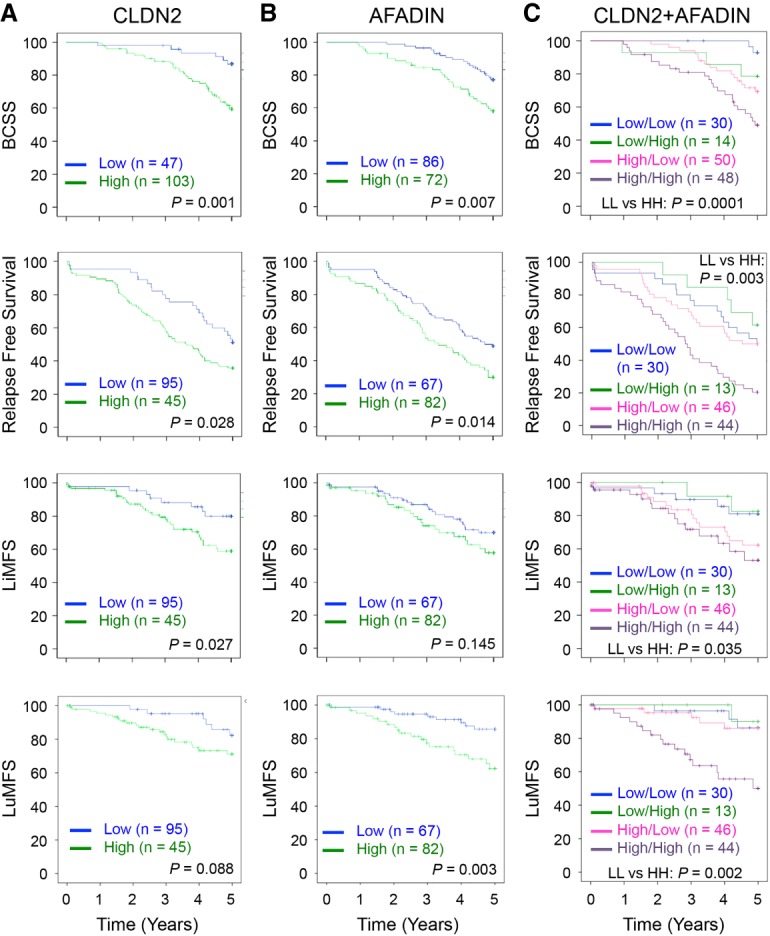
Claudin-2 and Afadin are prognostic of breast cancer liver metastasis. Kaplan-Meier analysis of metastatic breast cancer patients divided into high (green) and low (blue) reveals that expression of either Claudin-2 (*A*) or Afadin (*B*) is prognostic for BCSS and RFS. Claudin-2 expression is also prognostic of LiMFS (*A*), while Afadin expression is prognostic of LuMFS (*B*). (*C*) Further prognostic value is observed when considering Claudin-2^low^/Afadin^low^ (blue), Claudin-2^low^/ Afadin^high^ (green), Claudin-2^high^/Afadin^low^ (pink), and Claudin-2^high^/Afadin^high^ (purple) cohorts. (+) Censored.

We next assessed the association of Claudin-2 and Afadin expression either alone or in combination with four breast cancer end points: breast cancer-specific survival (BCSS), relapse-free survival (RFS), liver metastasis-free survival (LiMFS), or lung metastasis-free survival (LuMFS). As expected from the previous study, Kaplan-Meier analysis of 206 human metastatic breast cancer tumors revealed that high expression of Claudin-2 in primary breast tumors was associated with shorter BCSS (*P* = 0.001), an increased risk of developing distant metastases (*P* = 0.028), and liver-specific metastases (*P* = 0.027), although only a trend was observed with LuMFS (*P* = 0.088) (Fig. 6A). These results were also consistent in univariate Cox regression analyses using Claudin-2 continuous or dichotomized values (Supplemental Tables S1–S4). Dichotomized Claudin-2 values demonstrated a hazard ratio (HR) greater than any clinical parameters when assessing BCSS (*P* = 0.003, HR = 3.696, 95% CI 1.566–8.725) (Supplemental Table S1), RFS (*P* = 0.03, HR = 1.719, 95% CI 1.055–2.802) (Supplemental Table S2), or LiMFS (*P* = 0.032, HR = 2.346, 95% CI 1.074–5.123) (Supplemental Table S3). When combined with additional clinical parameters in multivariate analyses, Claudin-2 remains an independent prognostic factor for BCSS (*P* = 0.003, HR = 3.628, 95% CI 1.533–8.585) (Supplemental Table S1), RFS (*P* = 0.025, HR = 1.767, 95% CI 1.073–2.910) (Supplemental Table S2), and LiMFS (*P* = 0.047, HR = 2.214, 95% CI 1.010–4.853) (Supplemental Table S3).

In Kaplan-Meier analyses, high expression of Afadin was also significantly associated with poor patient prognosis when assessing BCSS (*P* = 0.007), RFS (*P* = 0.014), and LuMFS (*P* = 0.003), although only a trend was observed with LiMFS (*P* = 0.145) (Fig. 6B). These results were confirmed in univariate Cox regression analyses where dichotomized Afadin was associated with BCCS (*P* = 0.008, HR = 2.182, 95% CI 1.223–3.893) (Supplemental Table S1), RFS (*P* = 0.015, HR = 1.674, 95% CI 1.103–2.539) (Supplemental Table S2), and LuMFS (*P* = 0.005, HR = 3.137, 95% CI 1.418–6.941) (Supplemental Table S4) but did not reach significance for LiMFS (*P* = 0.148, HR = 1.555, 95% CI 0.855–2.830) (Supplemental Table S3). When added to additional clinical parameters, dichotomized Afadin remains independent from clinical parameters when using BCSS (*P* = 0.015 HR = 2.096, 95% CI 1.157–3.798) (Supplemental Table S1) and LuMFS (nodule: *P* = 0.005, HR = 3.106, 95% CI 1.404–6.873; size: *P* = 0.01, HR = 2.888, 95% CI 1.282-6.502) (Supplemental Table S4) and was close to significance when assessing RFS (*P* = 0.056 HR = 1.518, 95% CI 0.989–2.329) (Supplemental Table S2).

When considering both markers simultaneously, patients were stratified into four groups according to Claudin-2 and Afadin expression within the primary tumor: Claudin-2^low^/Afadin^low^, Claudin-2^low^/Afadin^high^, Claudin-2^high^/Afadin^low^, and Claudin-2^high^/Afadin^high^. Kaplan-Meier analysis revealed that high expression of both Claudin-2 and Afadin is associated with the worst prognosis for patients with respect to all four clinical end points (BCSS: *P* = 0.0001; RFS: *P* = 0.003; LiMFS: *P* = 0.035; LuMFS: *P* = 0.002) (Fig. 6C). Claudin-2 appeared to be the main driver for the observed correlation with LiMFS, as Afadin expression did not significantly confer a better or worse prognosis when compared with Claudin-2 alone. However, this was not the case for BCSS and RFS, where low Afadin expression in the context of low or high Claudin-2 expression displayed a positive impact on patient outcome (Fig. 6C). This was also reflected in multivariate Cox regression analyses when dichotomized Claudin-2 and Afadin were included in the analysis. Claudin-2 was independent from other parameters, while Afadin was not. This was observed for BCSS (Claudin-2: *P* = 0.008, HR = 3.615, 95% CI 1.406–9.297; Afadin: *P* = 0.061, HR = 1.832, 95% CI 0.972–3.453) (Supplemental Table S1) and RFS (Claudin-2: *P* = 0.038, HR = 1.751, 95% CI 1.033–2.969; Afadin: *P* = 0.12, HR = 1.445, 95% CI 0.908–2.299) (Supplemental Table S2).

## Discussion

In the present study, we implicate the C-terminal PDZ-binding motif as a critical determinant of Claudin-2 mediated growth in soft agar and liver metastasis. Claudin-2 has been shown to promote anchorage-independent growth of colorectal cancer cells, although the precise domains within Claudin-2 important for this function were not determined (Buchert et al. 2010; Dhawan et al. 2011). The ability of cancer cells to grow in an anchorage-independent fashion can promote their survival within the blood circulation and during early seeding events by providing resistance to anoikis (Gassmann and Haier 2008). Such a role is consistent with our previous observations that Claudin-2 provides an early survival advantage after seeding the liver (Tabariès et al. 2012). Given the importance of the PDZ-binding motif for growth in soft agar and the formation of liver metastases, it is conceivable that the PDZ-binding motif of Claudin-2 may also be required for early cancer cell survival within the liver.

Our previous studies demonstrated that breast cancer cells can adhere to hepatocytes through Claudin-2-dependent homotypic interactions (Tabariès et al. 2012). We show that the PDZ-binding motif within Claudin-2 is dispensable for the ability of breast cancer cells to adhere to hepatocytes. This is not surprising, as interactions between Claudin-2 expressed on breast cancer cells and Claudin-2 expressed in hepatocytes require the first extracellular loop of Claudin-2 (Tabariès et al. 2012). The ability of the Claudin-2 ΔPDZ BD mutant to rescue breast cancer cell/hepatocyte interactions indicates that this mutant localizes properly to the plasma membrane.

The importance of the PDZ-binding motif within the Claudin family for promoting metastasis and the identities of potential binding partners that interact through this region are still poorly understood. Current knowledge remains restricted to traditional PDZ domain-containing proteins. For example, the PDZ-binding motif of Claudin-1 has been shown to recruit Src in a complex with ZO-1 to confer resistance to anoikis in colon cancer (Singh et al. 2012). We interpret our data to mean that the PDZ-binding domain mutant of Claudin-2 is a loss-of-function mutant due to its inability to bind downstream effectors. However, it is conceivable that this mutant may function as a dominant negative to impede liver metastasis formation. Indeed, point mutations in claudins that prevent posttranslational modifications, which lead to protein stabilization, may exert dominant-negative effects on tight junction formation in cells with pronounced epithelial characteristics (D'Souza et al. 2005; Lee et al. 2005; Aono and Hirai 2008; Piehl et al. 2010). It has been reported that overexpression of Claudin-2 reduces tight junction integrity in MDCK I cells by interfering with claudins that form “tighter” tight junction complexes (Kondoh et al. 2008). However, to our knowledge, no posttranslational modifications within Claudin-2 have been reported to exhibit dominant-negative effects (Van Itallie et al. 2012a,b). It is important to note that the breast cancer models (4T1 and MDA-MB-231) used in this study have lost functional tight junctions by virtue of reduced expression of numerous tight junction components (Tabariès et al. 2011). In addition, of the claudins that we investigated, MDA-MB-231 cells express only Claudin-2 and Claudin-4. Importantly, we demonstrated previously that Claudin-4 is dispensable for liver metastasis (Tabariès et al. 2012). Together, these observations argue that the Claudin-2 ΔPDZ BD mutant may not function as a dominant negative to promote breast cancer liver metastasis.

In this study, we identified Pdlim7 and Afadin as new interacting partners of Claudin-2 that contribute to the ability of breast cancer cells to grow in soft agar and form liver metastases. Although little is currently known about potential roles that Pdlim7 may play in cancer tumorigenicity or metastasis, gene expression analysis of skin tumors identified *Pdlim7* to be more highly expressed in metastatic compared with nonmetastatic tumors (McCreery et al. 2015). Moreover, after comparing gene expression of breast cancer brain metastasis to bone metastasis, *Pdlim7* appeared to be specifically expressed in bone metastasis (Klein et al. 2009). Finally, Pdlim7 may play a role in promoting tumorigenesis by triggering mitosis and decreasing p53 antiproliferative activity (Jung et al. 2010). Kaplan-Meier survival analysis revealed that the expression level of Pdlim7 is associated with poor survival of breast cancer patients (Kales et al. 2014).

The concordant phenotypes exhibited by Claudin-2- and Afadin-deficient breast cancer cells suggest that a Claudin-2–Afadin signaling axis is important for the efficient formation of liver metastases. Our data also demonstrate that Claudin-2 and, to a greater extent, Afadin function as more general modulators of breast cancer metastasis to soft tissue sites, including the lung. Interestingly, Afadin has been demonstrated recently to interact with Claudin-6 in MDA-MB-231 breast cancer cells. Claudin-6 colocalized and interacted with Afadin, resulting in suppression of ERK signaling, increased stem cell characteristics, and enhanced chemoresistance to adriamycin (Yang et al. 2018).

Afadin is a ubiquitously expressed large F-actin-binding protein that mediates epithelial polarity (Mandai et al. 2013). It is located at adherens junctions, forming a large complex with adhesion proteins (e.g., nectins) and the actin cytoskeleton (Mandai et al. 1997, 2013; Ooshio et al. 2010). Afadin is also recruited to tight junctions via interactions with ZO-1 and claudins (Miyata et al. 2009; Ooshio et al. 2010; Mandai et al. 2013). Afadin exists as multiple isoforms that are generated by alternative splicing, which results in truncations near the C terminus of the protein (Carminati et al. 2016). Thus, in addition to full-length Afadin (l-Afadin), shorter isoforms that lack the F-actin domain (s-Afadin) are also expressed (Mandai et al. 2013; Carminati et al. 2016). The l-Afadin isoform is thought to be plasma membrane-associated and is not able to translocate to the nucleus, whereas the s-Afadin isoforms are dual-residency proteins that can shuttle from junctional complexes to the nucleus (Buchert et al. 2007). Nuclear-localized s-Afadin isoforms have been shown to regulate cell migration (Mandai et al. 2013; Carminati et al. 2016). We confirmed that both l-Afadin and s-Afadin isoforms are expressed in breast cancer cells. Claudin-2 and Afadin interact with one another predominantly in the membrane fraction, suggesting that l-Afadin might be important for growth in soft agar and formation of liver metastases. While the majority of Claudin-2 is localized to the membrane fraction, we did detect some Claudin-2 in the nucleus. Whether this simply represents contamination from other fractions or an actual signal will require additional experimentation. However, it has been reported that nuclear-localized Claudin-2 mediates cell proliferation in lung carcinoma cells (Ikari et al. 2014). Rescue experiments with both l-Afadin and s-Afadin isoforms were able to only partially restore the phenotype in Afadin-deficient breast cancer cells. This observation may suggest that Claudin-2 can mediate breast cancer liver metastasis not only through its interaction with Afadin but through Afadin-independent mechanisms as well. It is also conceivable that both the long and short isoforms must be coexpressed to completely restore Afadin-mediated liver metastases. To assess this possibility, rescue experiments using cell lines re-expressing both exogenous l-Afadin and s-Afadin isoforms will need to be performed.

An intact PDZ-binding motif in Claudin-2 is required for the observed interaction with Afadin in the membrane fraction. The simplest interpretation is that the Afadin PDZ domain directly interacts with the PDZ-binding motif within Claudin-2. However, it is possible that the interaction between Claudin-2 and Afadin is indirect and is bridged by ZO-1. It has been shown that ZO-1 can bind to Afadin, and this interaction is important for tight junction formation (Ooshio et al. 2010). Indeed, the SH3 domain of ZO-1 can interact with proline-rich regions 1 and 2 (PRR1/2) in Afadin (Mandai et al. 2013). In this way, ZO-1 can bind Claudin-2 (via the PDZ domain in ZO-1 and the PDZ-binding motif in Claudin-2) and recruit Afadin to the complex (via the SH3 domain in ZO-1 and the PRR1/2 in Afadin). Determining whether Claudin-2 directly or indirectly interacts with Afadin in liver metastatic breast cancer cells will require additional experimentation.

Our results confirm previous findings that Claudin-2 functions as a prognostic marker of breast cancer liver metastasis (Kimbung et al. 2014). Furthermore, we are the first to demonstrate that high Afadin expression serves as a biomarker associated with reduced BCSS and RFS. Coexpression of Claudin-2 and Afadin is associated with reduced BCSS, RFS, LiMFS, and LuMFS. The roles played by Afadin in the context of cancer are complex, with studies associating either tumor-suppressive or tumor-promoting roles to this adaptor protein (Letessier et al. 2007; Fournier et al. 2011; Sun et al. 2014; Yamamoto et al. 2015). Several studies have shown that loss of Afadin expression leads to enhanced cell invasion in diverse cancer types, including breast, colorectal, endometrial, and pancreatic cancer (Fournier et al. 2011; Sun et al. 2014; Xu et al. 2015; Yamamoto et al. 2015). In prostate cancer cells, Afadin expression inhibits proliferation and metastasis through down-regulation of Snail. Nuclear Afadin deficiency permits the formation of a Dishevelled 2 (Dvl2)–FOXE1 complex on the *Snail* promoter to activate its expression (Xu et al. 2015).

In contrast, Afadin has been shown to be important for heterotypic N-Cadherin/E-Cadherin interactions between cancer-associated fibroblasts and cancer cells that drive cellular invasion (Labernadie et al. 2017). Afadin can also contribute to chemoresistance in TNBC cells through interactions with Claudin-6 and subsequent suppression of ERK signaling (Yang et al. 2018). Roles for Afadin in modulating cell death and/or survival have also been reported. Afadin expression decreased apoptosis induced by serum deprivation or Fas ligand stimulation in cultured Afadin knockdown fibroblasts and endothelial cells compared with control cells. Indeed, Afadin knockdown in these cells impaired PDGF and/or VEGF-mediated activation of the phosphatidylinositol3-kinase (PI3K)–Akt signaling pathway, which is critically involved in cell survival (Kanzaki et al. 2008; Tawa et al. 2010). Independently, Afadin regulates cell proliferation by promoting VEGF-induced or sphingosine 1-phosphate-induced proliferation of endothelial cells (Tawa et al. 2010). Finally, our results support a role for Afadin, in cooperation with Claudin-2, in promoting the ability of breast cancer cells to metastasize to soft tissues such as the liver and lungs. The complex and context-dependent action of the Claudin-2/Afadin axis is reinforced by a recent study that describes the reactivation of ERK signaling pathway via the down-regulation of Afadin by Claudin-2, which decreases the migratory potential of osteosarcoma (OS) cells (Zhang et al. 2018). Moreover, reduced Claudin-2 and Afadin expression was associated with elevated pulmonary metastasis in OS patients (Zhang et al. 2018). Contrary to this, our study showed that coexpression of Claudin-2 and Afadin in primary breast tumors is associated with poor clinical outcomes, including increased risk of developing soft tissue-specific metastases (the liver and lungs). The precise molecular mechanisms through which Claudin-2 and Afadin contribute to these phenotypes require further experimentation and may shed light on the opposing roles of these cancers in different tumor types.

## Materials and methods

### Cell culture and transfections

The 4T1 and MDA-MB-231 cell lines were obtained from the American Type Culture Collection. MDA-MB-231TR cells transduced with a triple-reporter system were the kind gift of Dr. Joan Massagué (Minn et al. 2005). The generation of 4T1-derived liver-aggressive cell populations has been described previously (Tabariès et al. 2011). Expression of wild-type or a PDZ-binding motif mutant of Claudin-2 in breast cancer cells with reduced endogenous Claudin-2 was performed using an LMP vector system from a microRNA-30-adapted shRNAmir retroviral vector kit, adhering to the manufacturer's instructions (Open Biosystems) as described previously (Tabariès et al. 2012). The shRNA against *claudin-2* targets the 3′ untranslated region (UTR) and has the sequence 5′-CACACACAAGGTGATCAATAAA-3′.

The mutant form of mouse Claudin-2 harboring a deletion of the three C-terminal amino acids (GYV) was kindly provided by J.M. Anderson and has been described previously (Van Itallie et al. 2004). Wild-type and mutant Claudin-2 sequences were cloned into pEF1/HisB (Invitrogen) expression vectors. Transfections were performed using an Effectene kit (Qiagen, 301427). Stable cell lines were maintained in 1.5 µg/mL puromycin and/or 1.1 mg/mL G-418 antibiotic selection. Three individual clones expressing wild-type or mutant Claudin-2 were combined to generate pooled populations.

Claudin-2- or AF6-deficient MDA-MB-231TR cells were engineered using a CRISPR/Cas9 approach using the following single-guide RNA (*Claudin-2*: 5′-CACCGCACAAGTTGGAGGCC AAGAG-3′ and 5′-CACCGCTAGGCCTTCTGG GGCTTTT-3′; *AF6*: 3′-TATGGACGCAGAAACCTACG-3′) (Ran et al. 2013). The precise nature of the CRISPR/Cas9-mediated mutagenesis was verified by sequencing to ensure that each clone within the reconstituted pooled populations (Claudin-2^Crispr^ and Afadin^Crispr^) carried the expected gene disruptions. The HA-tagged versions of Claudin-2 were constructed by inserting a tandem HA tag into the intracellular loop after nucleotide 109 in the cDNA sequence of Claudin-2. The mouse cDNA sequences were then cloned into pMSCVpuro vectors (Clontech), while human cDNA sequences for *Claudin-2* were cloned into pQXCIB (Clontech) expression vectors. In order to express exogenous isoforms of Afadin, a cDNA encoding the short *Afadin* (*sAF6*) isoform was purchased from Dharmacon (Dharmacon, 100063579). cDNA was then shuttled into the pQCXIB retroviral expression vector. To obtain the long isoform of *Afadin* (*lAF6*), the C-terminal region of long human *Afadin* was PCR-amplified from MDA-MB-231 cells using a forward primer targeting exon 28 (3′-AGCGTTGGTATGAGAAGGAG-5′) and a reverse primer against the 3′ UTR (3′-CAAACTCGCACCTACAAACC-5′). The amplicon was transferred into pBluescript II KS(+) vector (Stratagene). The resulting plasmid was digested with RsrII/EcoRV and cloned into the Dharmacon vector containing the *sAF6* isoform to replace its C-terminal region. Finally, as for *sAF6*, the *lAF6* human isoform was inserted into the pQCXIB retroviral expression vector. Virus production and cell infection were performed as described previously (Tabariès et al. 2012). Mouse breast cancer cells were selected and maintained in 1.5 µg/mL puromycin (Invivogen, ant-pr), while human breast cancer cells were selected and maintained in 5 mg/mL blasticidin (Invivogen, ant-bl).

As reported previously, all lentiviral shRNA vectors were obtained from the arrayed Mission TRC genome-wide shRNA collections purchased from Sigma-Aldrich Corporation (Huang et al. 2012). Additional information describing the shRNA vectors is at http://www.sigmaaldrich.com/life-science/functional-genomics-and-rnai/shrna/library-information.html or http://www.broad.mit.edu/genome_bio/trc/rnai.html using the appropriate TRCN number. The following lentiviral shRNA vectors were used: *sh*mouse*AF6*, TRCN0000090484 and TRCN0000090486. Lentiviral supernatants were generated as described at http://www.broadinstitute.org/rnai/public/resources/protocols. Pooled stable populations were maintained under 1.5 µg/mL puromycin antibiotic selection.

### Anchorage-independent growth assay

Using 6-cm plates, 1 × 10^4^ 2776 liver-aggressive populations were seeded in 4 mL of 0.3% agar (BD Difco, 214220) in cell culture medium that was plated over a 6-mL layer of 0.6% agar. Five representative images per plate were captured using an AxioCam attached to an Axio Zoom.V16 microscope (Zeiss) using a 30× magnification, and the number of colonies was counted. The data represent the average of three independent experiments performed in triplicate.

### Experimental metastasis assays

For experimental liver metastasis assays, 1 × 10^5^ 2776 liver-aggressive cells or 1 × 10^6^ MDA-MB-231TR cells were injected into the spleens of 4- to 6-wk-old female Balb/c or NSG mice, respectively (Tabariès et al. 2011). Experimental lung metastasis assays were performed by injecting 5 × 10^5^ MDA-MB-231TR cells into the tail veins of NSG mice. Tumor burden in the liver or lungs was quantified using Imagescope software (Aperio) as described previously (Tabariès et al. 2011; Ngan et al. 2017). For tumor growth studies, 1 × 10^5^ cells were injected into the fourth mammary gland, and tumor volumes were determined as described previously (Rose et al. 2007).

The mice were housed in facilities managed by the McGill University Animal Resources Centre, and all animal experiments were conducted under a McGill University-approved Animal Use Protocol in accordance with guidelines established by the Canadian Council on Animal Care.

### Subcellular fractionation

Subcellular fractionation was performed as per the manufacturer's instructions using the subcellular protein fractionation kit for cultured cells (Thermo Scientific, 78840). Briefly, the cytoplasmic extract was prepared by lysing the cell pellet in cytoplasmic extract buffer (150 mM NaCl, 50 mM HEPES at pH 8.0, 100 µg of digitonin) in the presence of protease inhibitors. The membrane extract was isolated using mass spectrometry lysis buffer (M.S.: 100 mM KCl, 50 mM HEPES at pH 8.0, 0.1% NP-40, 2 mM EDTA at pH 8.0, 10% glycerol) with protease inhibitors. The nuclear extract was then isolated in TNE lysis buffer as described previously (Siegel et al. 1999). Each fraction was analyzed by immunoblotting.

### Immunoblotting

Cell lysates were generated and membranes were processed as described previously (Tabariès et al. 2011). Immunoblot analyses were performed using the following antibodies: Claudin-2 (1:5000; Thermo Fisher, 325600), AF6 (1:5000; BD Transduction, 610732), EGFR (1:5000; Epitomics, 1902-1), LaminA/C (1:5000; Cell Signaling, 4777), α-Tubulin (1:10,000; Sigma, T9026), and HA.11 clone 16B12 (1:10,000; Covance, MMS-101-P-200) antibodies. The Pdlim7 antibody was generated in the laboratory of Dr. Hans-Georg Simon as described previously (Camarata et al. 2006). Membranes were incubated with their corresponding horseradish peroxidase (HRP)-conjugated anti-IgG secondary antibodies (Jackson ImmunoResearch Laboratories, Inc.) or Amersham ECL antimouse IgG horseradish peroxidase-linked species-specific whole antibody (GE Healthcare) and visualized with chemiluminescent HRP substrate (Millipore, WBLUF0500) or an enhanced chemiluminescence system (Thermo Fisher, 34578).

### Immunoprecipitation

The membrane fractions generated by subcellular fractionation were quantified using a Bradford assay. In 1-mL aliquots, 0.7–1.0 mg of protein was used for each immunoprecipitation, and the lysate was precleared for 30 min using 20 µL of 50% protein G-Sepharose (GE Bioscience). Using 0.75–3 µg of either Claudin-2 (Life Sciences, 516199 ), Afadin (Cell Signaling, 13531S), or isotype control IgG (Jackson ImmunoResearch, 011-000-003) antibodies, the aliquots were incubated along with 50 µL of 50% sepharose beads. For HA immunoprecipitation, anti-HA affinity matrix (Roche, 11-015-010-001) was used. Samples were incubated under rotation in the cold room for 2 h and subsequently washed in lysis buffer and ammonium bicarbonate (pH 7). Hybridized beads were suspended in sodium dodecyl sulfate buffer with 15% 2-mercaptoethanol. Samples were then processed for immunoblot analysis.

### Patients, TMA, and immunohistochemistry

Patient cohorts and TMA construction were described previously (Kimbung et al. 2014). Clinical and pathological characteristics of the cohort are listed in Supplemental Table S5.

Immunohistochemical staining was performed using a Claudin-2 mouse monoclonal antibody (Thermo Fisher Scientific, clone 12H12) at a 1:250 dilution. For Afadin, a rabbit polyclonal antibody at a dilution of 1:300 was used (Sigma, HPA030212). Serial TMA sections were stained using a Discovery Ultra autostainer (Ventana Medical System, Inc.) following the manufacturer's instructions. As observed previously, Claudin-2 staining was detected as a membranous and cytoplasmic granular reaction (Tabariès et al. 2011, 2012; Kimbung et al. 2014). Afadin staining was detected mostly at the membrane and to a lesser extent in the cytoplasm. Each sample was given a semiquantitative score from 0 to 4 for the proportion of tumor cells staining positive (1 [<25%], 2 [<25% or >50%], 3 [<50% or >75%], and 4 [>75%]) and from 0 to 3 for the intensity of tumor cell staining (0 [no staining], 1 [weak], 2 [moderate], and 3 [strong]). The proportion and intensity scores were combined by multiplication to obtain a final weighted-score ranging from 0 to 12. Two independent reviewers performed scoring.

### Statistical analysis

Significance values associated with differences in anchorage-independent growth assays and those associated with liver or lung metastasis formation (Figs. 1–4) were calculated using a Student's *t*-test. In Figure 6, the median expression of Claudin-2 or Afadin was used to dichotomize the data for Kaplan-Meier analyzes. Thus, a weighted-score ≥2 was considered as a high expression score for Claudin-2, while a weighted score ≥3 was considered as a high expression score for Afadin. BCSS, RFS, LiMFS, and LuMFS curves were plotted using the Kaplan-Meier estimator, and the log-rank test was used to evaluate significant differences. Univariate and multivariate proportional hazard model (Cox regression) was used to estimate the HRs (Supplemental Tables S1–S4). Since the number of events did not allow more than three or four parameters into the model, we used the N and T stages for multivariate analyses, as they were the only two parameters significant for LiMFS or LuMFS. All *P*-values correspond to two-sided statistical tests, and values <0.05 were considered significant. The statistical software package IBM SPSS Statistics 25 (IBM Corporation) was used.

## Supplementary Material

Supplemental Material
