# Supplementary material for: Afadin cooperates with Claudin-2 to promote breast cancer metastasis
Source: Genes Dev. 2019 Feb 1;33(3-4):180–93. doi: 10.1101/gad.319194.118 (PMC6362814; doi:10.1101/gad.319194.118)
Supplement: Supplemental Material [file supp_33_3-4_180__index.html]

Afadin cooperates with Claudin-2 to promote breast cancer metastasis — Supplemental Material 

# Afadin cooperates with Claudin-2 to promote breast cancer metastasis

## Supplemental Material

- Supplemental\_Figure\_S1.pdf
- Supplemental\_Information.docx
- Supplemental\_Table\_S4.pdf
- Supplemental\_Figure\_S6.pdf
- Supplemental\_Table\_S2.pdf
- Supplemental\_Figure\_S4.pdf
- Supplemental\_Figure\_S2.pdf
- Supplemental\_Table\_S1.pdf
- Supplemental\_Table\_S5.pdf
- Supplemental\_Figure\_S7.pdf
- Supplemental\_Table\_S3.pdf
- Supplemental\_Figure\_S5.pdf
- Supplemental\_Figure\_S3.pdf
- Supplemental\_Table\_S6.pdf
