# Supplementary figures and images for: Afadin cooperates with Claudin-2 to promote breast cancer metastasis
Source: Genes Dev. 2019 Feb 1;33(3-4):180–93. doi: 10.1101/gad.319194.118 (PMC6362814; doi:10.1101/gad.319194.118)

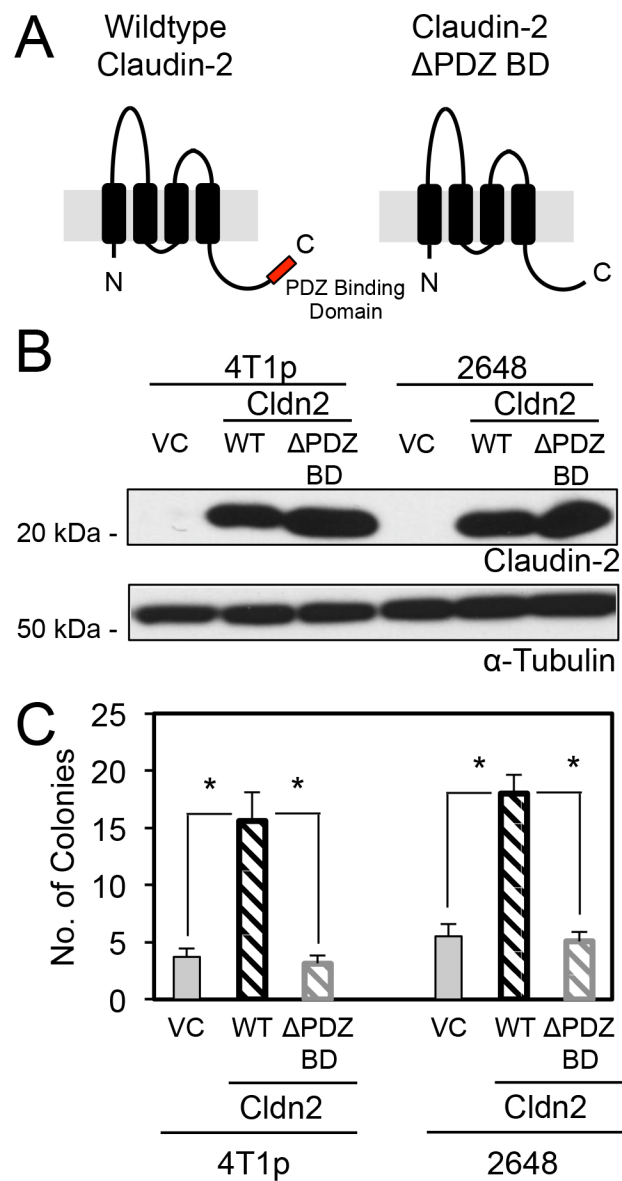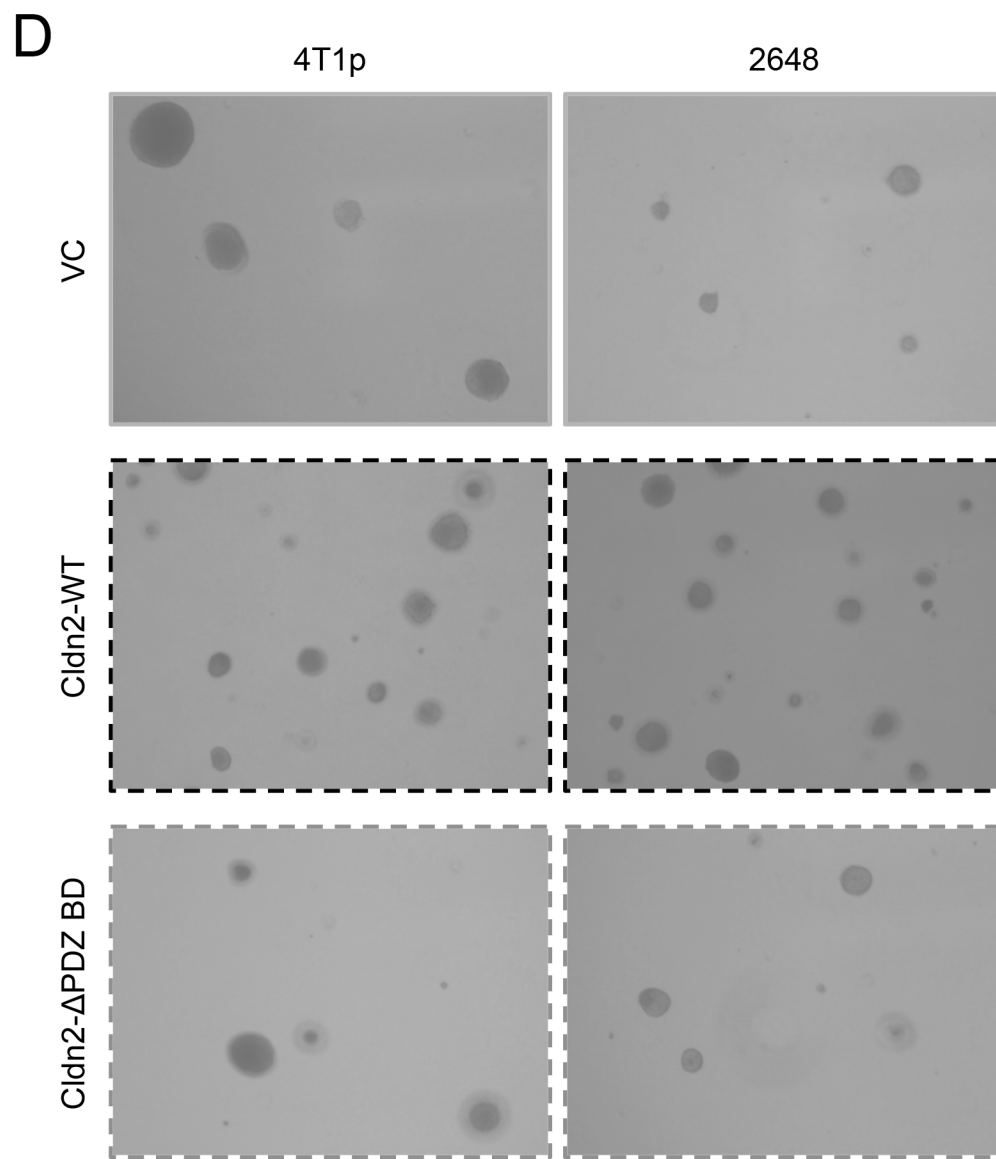

Supplement: Supplemental Material [file supp_gad.319194.118_Supplemental_Figure_S1.pdf]

**A**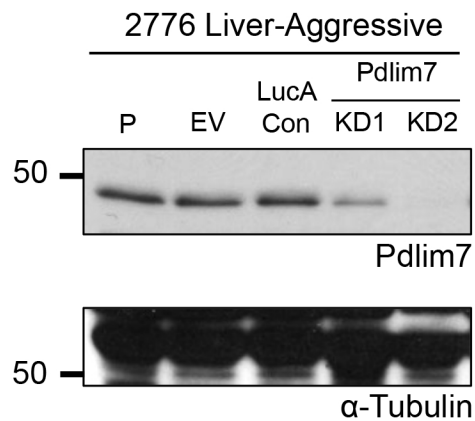**B**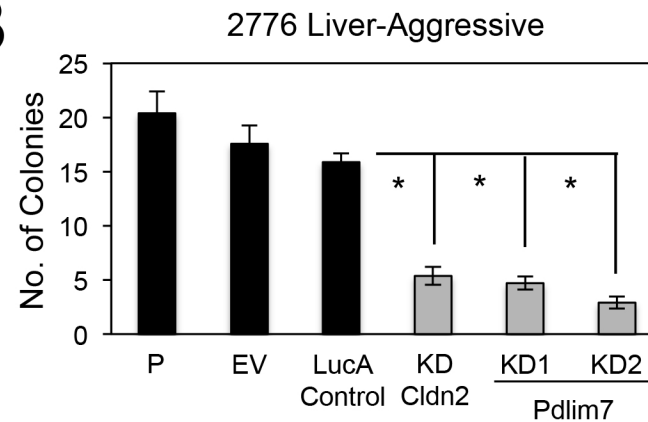**C**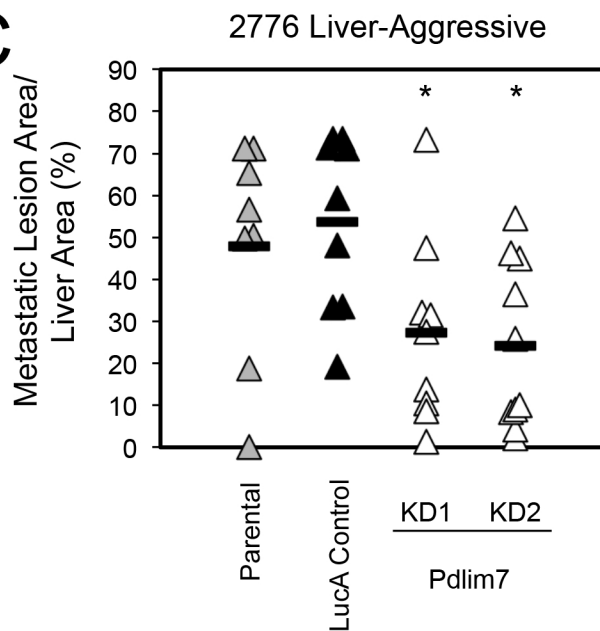**D**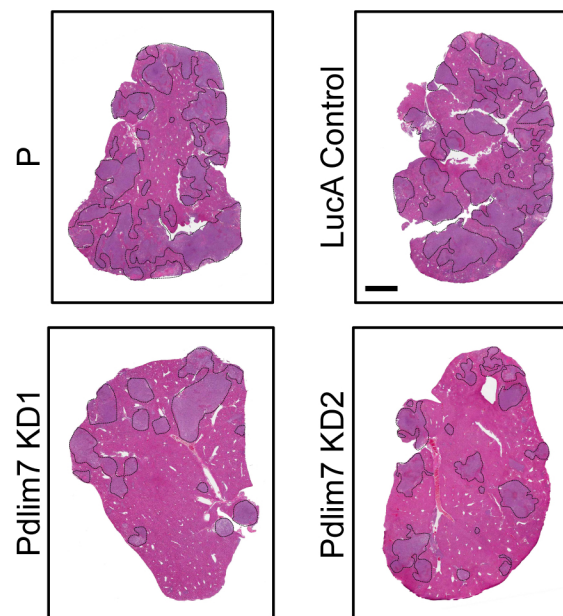

Supplement: Supplemental Material [file supp_gad.319194.118_Supplemental_Figure_S6.pdf]

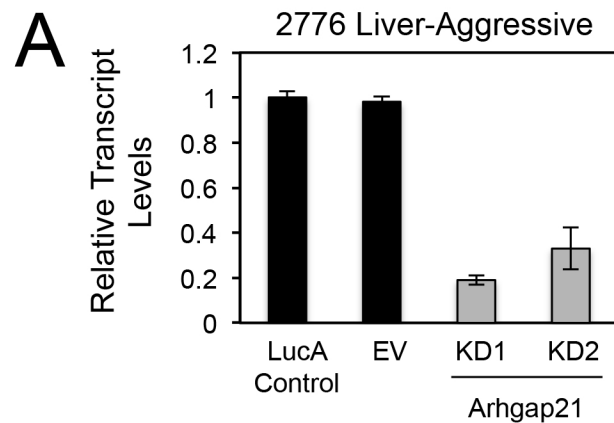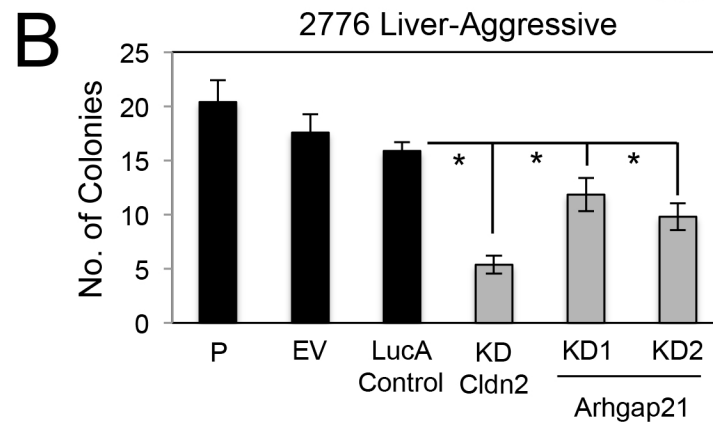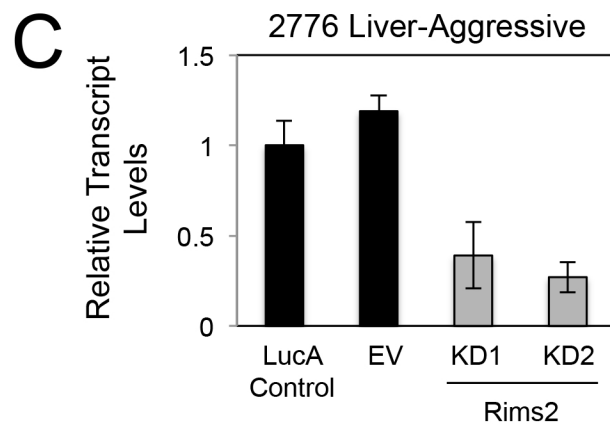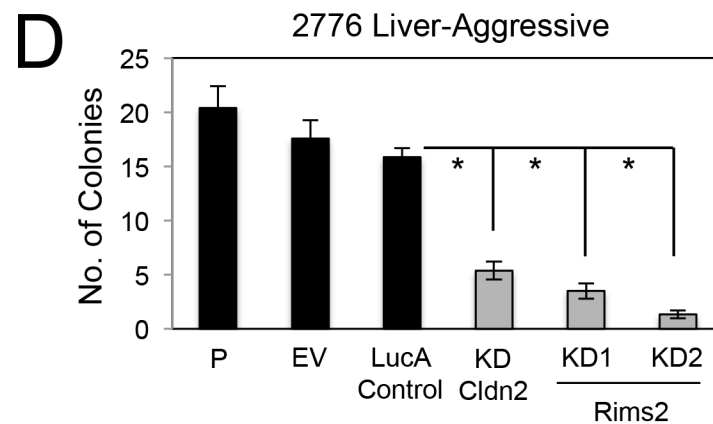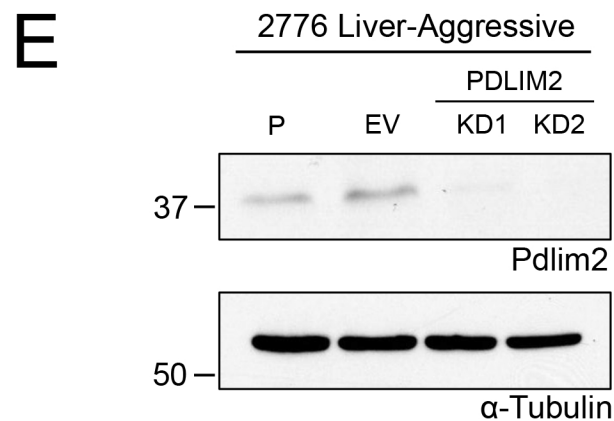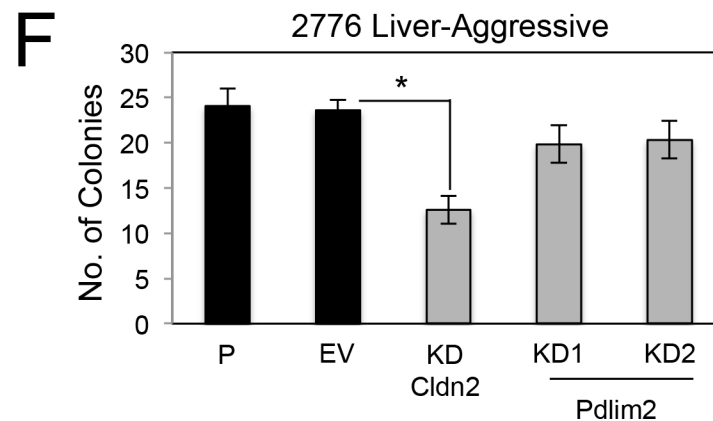

Supplement: Supplemental Material [file supp_gad.319194.118_Supplemental_Figure_S4.pdf]

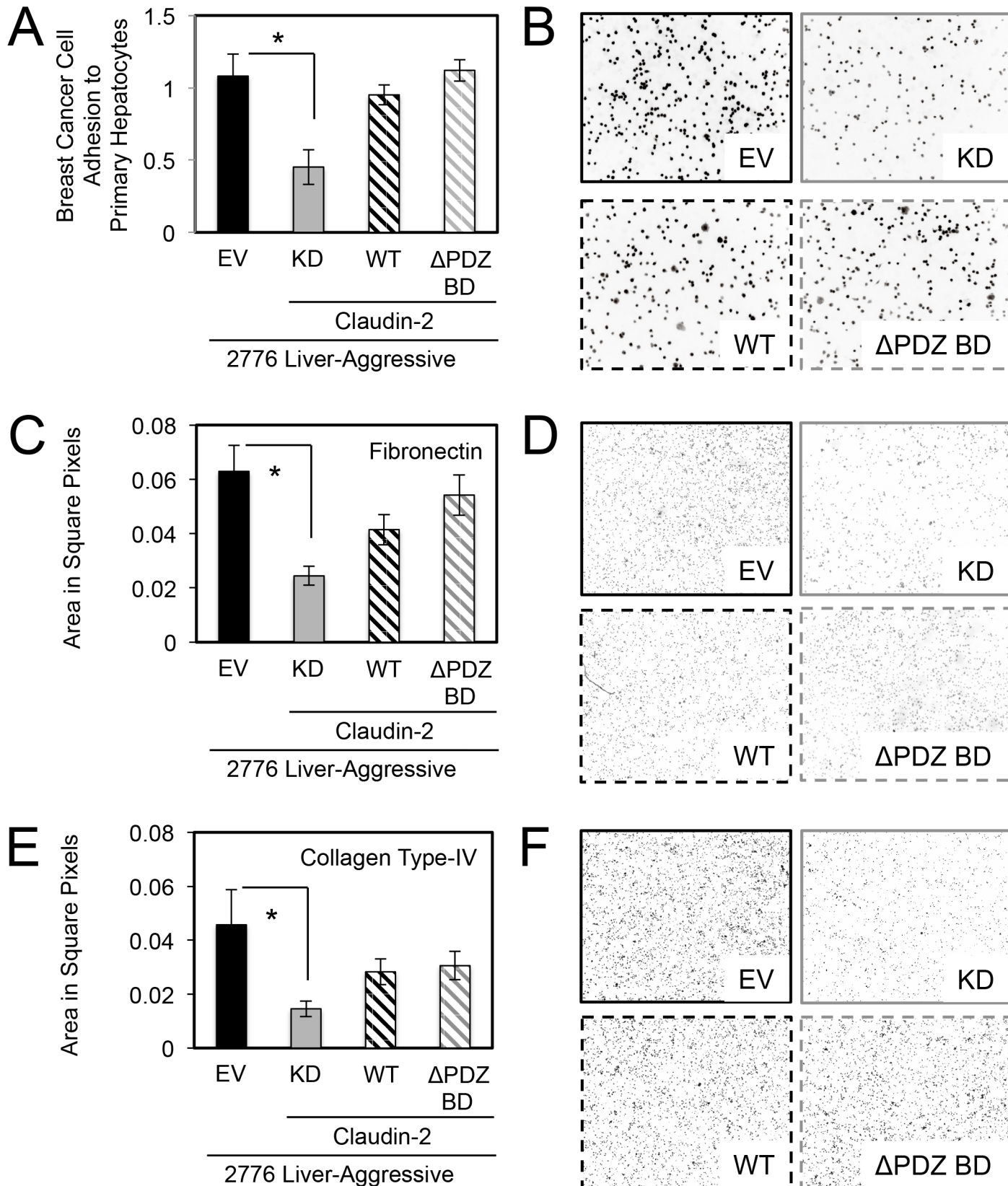

Supplement: Supplemental Material [file supp_gad.319194.118_Supplemental_Figure_S2.pdf]

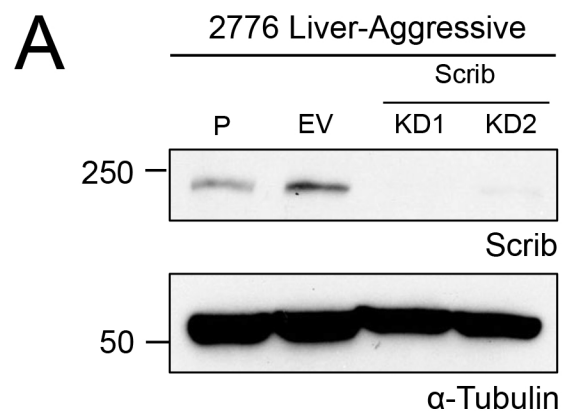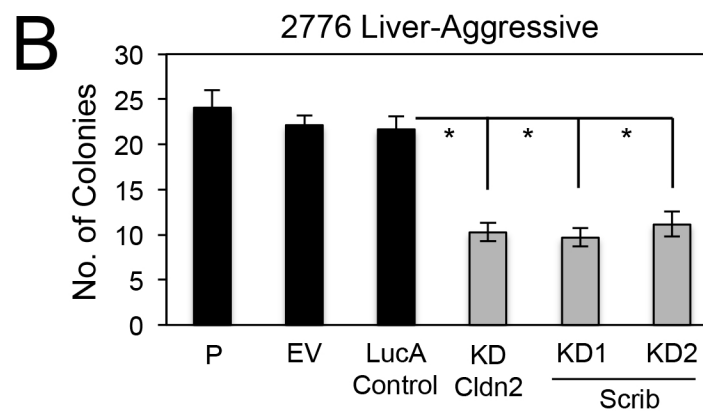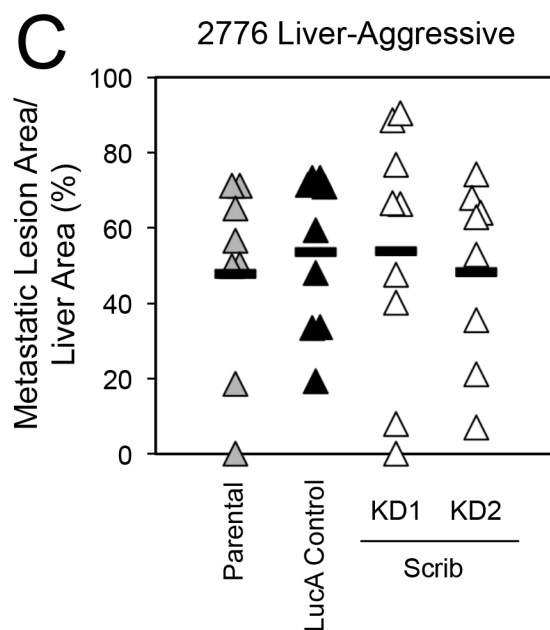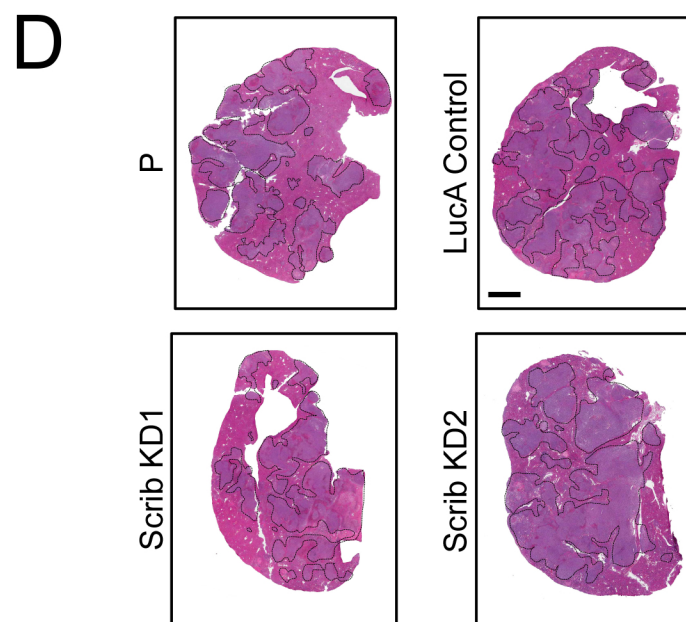

Supplement: Supplemental Material [file supp_gad.319194.118_Supplemental_Figure_S5.pdf]

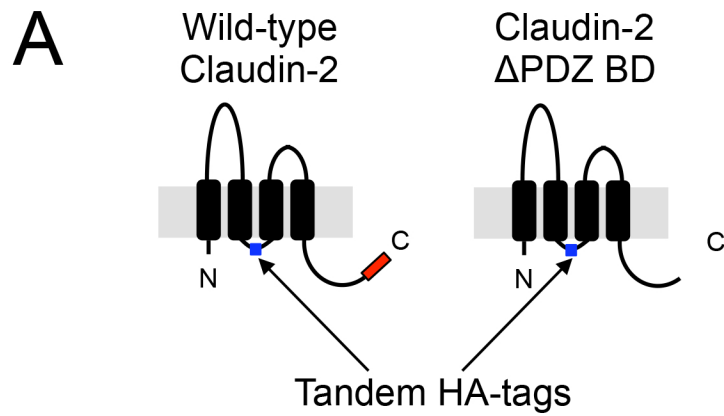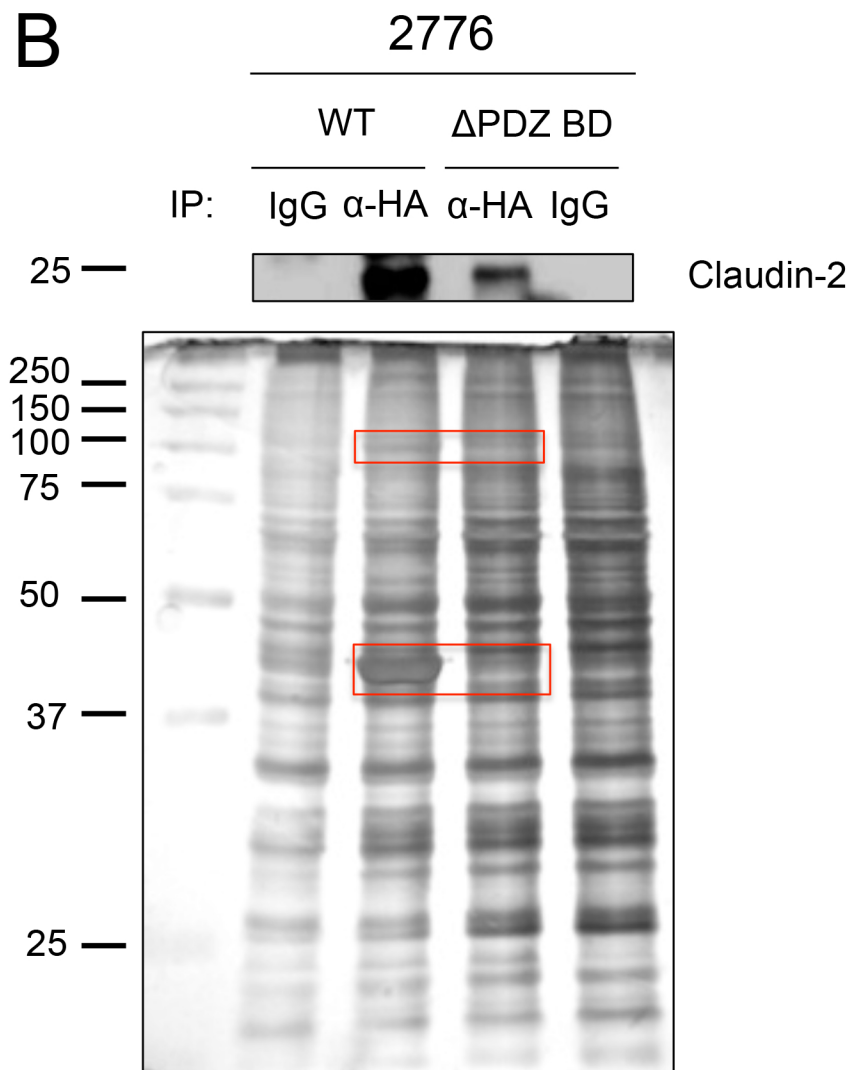

Supplement: Supplemental Material [file supp_gad.319194.118_Supplemental_Figure_S3.pdf]
