# Supplementary material for: Afadin cooperates with Claudin-2 to promote breast cancer metastasis
Source: Genes Dev. 2019 Feb 1;33(3-4):180–93. doi: 10.1101/gad.319194.118 (PMC6362814; doi:10.1101/gad.319194.118)
Supplement: Supplemental Material [file supp_gad.319194.118_Supplemental_Information.docx]

**Supplemental Information**

**Supplemental Materials and Methods**

***Cell Culture***

All lentiviral shRNA vectors were obtained from the arrayed Mission**^®^** TRC genome-wide shRNA collections purchased from Sigma-Aldrich Corporation (Huang et al. 2012). Additional information describing the shRNA vectors can be found at http://www.sigmaaldrich.com/life-science/functional-genomics-and-rnai/shrna/library-information.html or http://www.broad.mit .edu/genome_bio/trc/rnai.html, using the indicated TRCN numbers. The following lentiviral shRNA vectors were used: mouse *shArhgap21*: TRCN0000252194 and TRCN0000252185; mouse *shRims2*: TRCN0000106155 and TRCN0000106157; mouse *shPdlim2:* TRCN0000108840 and TRCN0000108841; mouse *shPdlim7*: TRCN0000178443 and TRCN0000375190; and mouse *shScrib*: TRCN0000111125 and TRCN0000111128. Lentiviral supernatants were generated as described at http://www.broadinstitute.org/rnai/public/ resources/protocols. Pooled stable populations were maintained in 1.5µg/ml puromycin antibiotic selection.

***Immunoblotting***

Cell lysates were generated and membranes were processed as previously described (Tabariès et al. 2011). Immunoblot analyses were performed using the following antibodies: Pdlim2 (1:5,000; Cat. #: 8144S, Cell Signaling), Pdlim7 (1:5,000; kind gift from Dr. Hans-Georg Simon) and Scrib (1:5,000; Cat. #: 4475S, Cell Signaling). Membranes were incubated with the appropriate Horseradish-peroxidase-conjugated anti-IgG secondary antibodies (Jackson ImmunoResearch Laboratories, Inc.) or with Amersham ECL Anti-Mouse IgG, Horseradish Peroxidase-Linked Species-Specific Whole Antibody (GE Healthcare) and visualized with chemiluminescent HRP Substrate (Cat. #: WBLUF0500, Millipore) or an enhanced chemiluminescence system (Cat. #: 34578, Thermofisher).

***Cell attachment assays***

The ability of 2776 liver-aggressive-derived cells to adhere to fibronectin or type IV collagen was assessed as described previously by seeding 1x10^5^ cells onto pre-coated 24-well plates (Cat. #: 354411 and 354430, BD Biosciences) (Tabariès et al. 2011).

***Hepatocyte adhesion assay***

Hepatocyte adhesion assays were performed as previously described (Tabariès et al. 2012). Briefly, 1 x 10^5^ carboxyfluorescein diacetate, succinimidyl ester (CFDA SE; Cat. #: V12883, Invitrogen)–labeled cells were seeded onto a monolayer of primary hepatocytes. Cells were incubated for 1h at 37°C, washed twice with PBS and fixed in 10% formalin (Fisher Scientific) for 20 minutes. Cells were visualized using epifluorescence after 2 washes in PBS. Two independent experiments were performed to generate the data using 4 wells/experiment and 5 fields/well.

***Silver staining***

Silver staining protocol compatible with mass spectrometry was last revised by C. Pomiès and kindly provided by P. Thibault. First, the gel was fixed (50% ethanol, 5% acetic acid) for 30 minutes and subsequently incubated in 50% ethanol at 4°C overnight. The gel was washed three times in water for 5 minutes/wash, sensitized in 0.02% sodium thiosulfate for 1 minute and subjected to 3 x 30 second washes in water. The gel was developed in 0.05% formalin, 3% sodium carbonate. Staining was stopped in 5% acetic acid for 5 minutes and the gel stored in 1% acetic acid at 4°C.

***RT-qPCR***

Total RNA was extracted from the indicated cell populations using RNeasy Mini Kits and QIAshredder columns (Cat. #: 74106 and 79656, Qiagen). According to manufacturer’s protocol, 1 μg of total RNA was converted to DNA using a High Capacity cDNA Reverse Transcription Kit (Cat. #: 4368813, Applied Biosystems). Following the reverse transcription (RT) reaction, all samples were diluted 1:50 in ddH_2_O and subjected to real time PCR analysis with SYBR Green PCR Master Mix (Cat. #: 4309155, Applied Biosystems). Ten picograms of gene specific primers (Supplementary Table S6) were used in a total reaction volume of 15 μl. For all targets, the cycling conditions were: 95°C for 10 minutes, followed by 40 cycles each consisting of 95°C for 15 seconds, 60°C for 30 seconds and 72°C for 45 seconds. Incorporation of SYBR Green dye into the PCR products was monitored using a 7500 Real time PCR system (Applied Biosystems). Serial dilutions were performed to generate a standard curve for each gene target in order to define the efficiency of the RT-qPCR reaction. The integrity and specificity of the amplified PCR products were confirmed by dissociation curve analysis (SDS 2.0 software, Applied Biosystems). Pfaffl analysis method was used to measure the relative quantity of gene expression (Pfaffl 2001). The reference gene, *Gapdh*, was selected based on its stable expression in all cell populations analyzed. Relative mRNA levels were expressed in terms of fold induction over the LucA control cell population. All measurements were done in triplicate and three independent experiments were performed for each gene target.

***Statistical Analysis***

Significance values associated with differences in anchorage independent growth assays, adhesion to hepatocytes, type IV collagen or fibronectin and those associated with liver metastasis formation (Figures S1, S2, S4, S5, S6) were calculated using a Student's *t*-test. In Figure S7, *P* values in panel B and C were obtained using the non-parametric Mann-Whitney U test and values **<**0.05 were considered significant. The statistical software package IBM SPSS Statistics 25 (IBM Corporation, NY) was used. *AFDN* expression, copy number, and clinical data was downloaded from the cbioportal (http://www.cbioportal.org) on Sept 20, 2018. Z-scores were generated by computing the relative expression of *AFDN* to the *AFDN* expression distribution in a reference population defined as all samples that are diploid at the *AFDN* locus. Z-scores therefore indicate the number of standard deviations away from the mean of expression in the reference population. Analysis was done using GraphPad Prism 7 Software.

**Supplementary References**

Huang S, Holzel M, Knijnenburg T, Schlicker A, Roepman P, McDermott U, Garnett M, Grernrum W, Sun C, Prahallad A et al. 2012. MED12 controls the response to multiple cancer drugs through regulation of TGF-beta receptor signaling. *Cell* **151**: 937-950.

Pfaffl MW. 2001. A new mathematical model for relative quantification in real-time RT-PCR. *Nucleic Acids Res* **29**: e45.

Tabariès S, Dong Z, Annis MG, Omeroglu A, Pepin F, Ouellet V, Russo C, Hassanain M, Metrakos P, Diaz Z et al. 2011. Claudin-2 is selectively enriched in and promotes the formation of breast cancer liver metastases through engagement of integrin complexes. *Oncogene* **30**: 1318-1328.

Tabariès S, Dupuy F, Dong Z, Monast A, Annis MG, Spicer J, Ferri LE, Omeroglu A, Basik M, Amir E. 2012. Claudin-2 promotes breast cancer liver metastasis by facilitating tumor cell interactions with hepatocytes. *Molecular and cellular biology* **32**: 2979-2991.

**Supplemental Figure Legends**

**Supplemental Figure S1. The PDZ-binding motif of Claudin-2 is required breast cancer cell anchorage independent growth.** **(A)** Schematic of wild-type and the ΔPDZ BD mutant of Claudin-2. **(B)** Immunoblot analysis of Claudin-2 expression in the indicated cell populations. An immunoblot for α-Tubulin served as a loading control. **(C)** The indicated cell populations were seeded in soft agar and the resulting colonies quantified (*, *P* < 0.0002). **(D)** Representative images of colonies formed by the indicated cell populations are shown.

**Supplemental Figure S2. The PDZ-binding motif of Claudin-2 is dispensable for Claudin-2 mediated adhesion of breast cancer cells to hepatocytes, fibronectin or collagen IV.** **(A)** The indicated cell populations were plated onto monolayers of primary hepatocytes and adhesion was quantified after 1h (*, *P* = 0.0001). **(B)** Representative images of each cell population following adhesion to a monolayer of primary hepatocytes are shown. **(C)** The indicated cell populations were plated onto fibronectin, the adhered cells were quantified and the data presented in pixels per unit area (*, P ≤ 0.031). **(D)** Representative images of the indicated cell populations following adhesion to fibronectin are shown. **(E)** Adhesion of the indicated cell populations to collagen IV was quantified and presented in pixels per unit area. Significant changes in adhesion to collagen IV are indicated (*, *P* ≤ 0.0033). **(F)** Representative images of the indicated cell populations adhered to collagen IV are shown.

**Supplemental Figure S3. Immunoprecipitation of HA-tagged Claudin-2 constructs reveals potential binding partners that interact via the PDZ-binding motif of Claudin-2.** **(A)** Schematics depicting HA-tagged wild-type and the ΔPDZ-binding motif mutant of Claudin-2. The HA-tag is located in the intracellular loop of Claudin-2. **(B)** Immunoblot analysis of HA-tagged wild-type and Claudin-2 ΔPDZ BD following immunoprecipitation using anti-HA antibodies. Anti-HA or IgG isotype control immunoprecipitates were separated by SDS-PAGE and visualized by silver staining. A representative image of a stained gel is shown. Differential band intensities were observed between the wild-type and ΔPDZ BD mutant of Claudin-2 (red boxes).

**Supplemental Figure S4. Arhgap21 and Rims2 expression is required for colony formation of breast cancer cells in soft agar.** **(A)** Real time - quantitative PCR (RT-qPCR) analysis was performed to measure *Arhgap21* expression (normalized to total *Gapdh* levels) in the indicated cell populations. The data is depicted as fold expression relative to control and is representative of 3 independent experiments performed in triplicate. **(B)** The indicated cells lines were plated in soft agar and the resulting colonies quantified (*, *P* < 0.0001). **(C)** RT-qPCR analysis was performed to measure *Rims2* expression (normalized to total *Gapdh* levels) in the indicated cell populations. The data is depicted as fold expression relative to control and is representative of 3 independent experiments performed in triplicate. **(D)** The indicated cells lines were plated in soft agar and the resulting colonies quantified (*, *P* < 0.00001). **(E)** Pdlim2 expression in the indicated cell populations was assessed by immunoblot, with α-Tubulin serving as a loading control. **(F)** The indicated cells lines were plated in soft agar and the resulting colonies quantified (*, *P* < 8x10^-18^).

**Supplemental Figure S5. Scrib expression is required for anchorage-independent growth but dispensable for breast cancer liver metastasis.** **(A)** Immunoblot analysis of Scrib expression in the indicated cell populations. An immunoblot for α-Tubulin served as a loading control. **(B)** Colony formation in soft agar was quantified for the indicated cell populations (*, *P* < 0.00001). **(C)** Liver-metastatic burden (tumor area/tissue area) was quantified following splenic injection of the indicated cell lines. **(D)** Representative images of the cardiac liver lobe for the indicated cell populations are shown. Metastatic lesions are outlined by dotted lines. Scale bar represents 2mm and applies to all panels.

**Supplemental Figure S6. Pdlim7 expression promotes anchorage independent growth and breast cancer liver metastasis. (A)** Immunoblot analysis of Pdlim7 expression in the indicated cell populations. Immunoblotting for α-Tubulin served as a loading control. **(B)** Colony formation in soft agar was quantified for the indicated cell populations (*, *P* < 0.00001). **(C)** Liver-metastatic burden (tumor area/tissue area) was quantified following splenic injection of the indicated cell lines (*, *P* < 0.01 compared to LucA control). **(D)** Representative images of the cardiac liver lobe for each cell population are shown. Metastatic lesions are outlined by dotted lines. Scale bar represents 2mm and applies to all panels.

**Supplemental Figure S7. Claudin-2 and Afadin are prognostic of breast cancer liver metastasis.** **(A)** Tissue microarray of 206 metastatic breast cancer primary tumors metastases was subjected to immunohistochemical staining with an anti-claudin-2 antibody or an anti-Afadin antibody. Claudin-2 and Afadin staining was scored by two independent reviewers. Representative images for each intensity category are shown. Scale bar represents 50μm. Immunohistochemical staining analysis reveals that higher expression levels of either Claudin-2 **(B)** or Afadin **(C)** are associated with TNBC compared to ER+ (**, *P* = 0.002 and *P* < 0.001, respectively). Claudin-2, but not Afadin, expression is also higher in TNBC compared to HER2 subtypes (***, *P* = 0.01 and 0.173, respectively). **(D)** *Afadin* expression stratified by histological subtype. Horizontal bars indicate mean *AFDN* expression (*, *P* = 0.0001). **(E)** *AFDN* mRNA expression in breast cancer patients stratified by copy number status, highlighting patients expressing high levels of *AFDN;* Z>0 (average expression above the mean expression on *AFDN* in diploid tumors). Horizontal bars indicate mean *AFDN* expression. **(F)** Kaplan Meier analysis of breast cancer patient survival stratified by high versus low *AFDN* mRNA expression. *AFDN-*high was defined as patients with *AFDN* expression above the mean *AFDN* expression in diploid tumors; Z>0, n=1091 patients in total. Homdel=homozygous deletion, Het Loss=loss of heterozygosity, Amp=amplification, HR=hazard ratio, ER+=estrogen receptor positive, HER2+= human epidermal growth factor receptor 2 positive, TNBC=triple negative breast cancer.
