# Supplementary material for: Afadin cooperates with Claudin-2 to promote breast cancer metastasis
Source: Genes Dev. 2019 Feb 1;33(3-4):180–93. doi: 10.1101/gad.319194.118 (PMC6362814; doi:10.1101/gad.319194.118)
Supplement: Supplemental Material [file supp_gad.319194.118_Supplemental_Table_S4.pdf]

Supplemental Table S4: 5-years lung metastasis free survival (LuMFS).

	Univariate				Multivariate*				Multivariate**			
	P	HR	95.0% CI for HR		P	HR	95.0% CI for HR		P	HR	95.0% CI for HR	
			Lower	Upper			Lower	Upper			Lower	Upper
Age (>50 vs<=50)	0.144	1.638	0.846	3.174	-	-	-	-	-	-	-	-
ER (+ vs -)	**<0.001**	0.25	0.125	0.498	-	-	-	-	-	-	-	-
Grade TMA (3 vs 1-2)	**0.011**	2.704	1.259	5.808	-	-	-	-	-	-	-	-
Nodule (N+vsN0)	0.256	1.488	0.749	2.956	0.646	1.208	0.538	2.713	-	-	-	-
Size (>2cm vs <=2cm)	**0.007**	2.626	1.299	5.311	-	-	-	-	0.069	2.170	0.942	4.997
Claudin-2_Continuous	**0.004**	1.254	1.077	1.461	**0.005**	1.244	1.067	1.45	**0.008**	1.229	1.055	1.432
Afadin_Continuous	**0.001**	1.198	1.074	1.336	**0.001**	1.207	1.081	1.348	**0.002**	1.186	1.062	1.324
Claudin-2 Low-High	0.096	2.163	0.872	5.368	0.100	2.146	0.864	5.326	0.093	2.183	0.878	5.423
Afadin Low-High	**0.005**	3.137	1.418	6.941	**0.005**	3.106	1.404	6.873	**0.010**	2.888	1.282	6.502

Abbreviations: HR, Hazards Ratio; CI, confidence interval; ER, estrogen receptor.
Numbers in bold represent statistically significant differences.
*Each marker was added one at the time in the model with clinical parameter (N stage). Results of the clinical parameters were those when associated with Claudin-2 continuous
**Each marker was added one at the time in the model with clinical parameter (T stage). Results of the clinical parameters were those when associated with Claudin-2 continuous
