# Supplementary material for: Afadin cooperates with Claudin-2 to promote breast cancer metastasis
Source: Genes Dev. 2019 Feb 1;33(3-4):180–93. doi: 10.1101/gad.319194.118 (PMC6362814; doi:10.1101/gad.319194.118)
Supplement: Supplemental Material [file supp_gad.319194.118_Supplemental_Table_S2.pdf]

Supplemental Table S2: 5-years relapse-free survival (RSF).

	Univariate				Multivariate*				Multivariate**			
	P	HR	95.0% CI for HR		P	HR	95.0% CI for HR		P	HR	95.0% CI for HR	
			Lower	Upper			Lower	Upper			Lower	Upper
Age (>50 vs<=50)	**<0.001**	2.018	1.369	2.975	-	-	-	-				
ER (+ vs -)	**<0.001**	0.434	0.280	0.672	-	-	-	-				
Grade TMA (3 vs 1-2)	**0.004**	1.806	1.213	2.687	-	-	-	-				
Nodule (N+vsN0)	**0.001**	2.070	1.357	3.157	**0.045**	1.675	1.011	2.775	0.063	1.641	0.974	2.764
Size (>2cm vs <=2cm)	**<0.001**	2.318	1.566	3.432	**0.004**	2.032	1.257	3.285	0.014	1.882	1.136	3.118
Claudin-2_Continuous	**0.003**	1.157	1.052	1.273	**0.008**	1.136	1.033	1.248				
Afadin_Continuous	**0.041**	1.075	1.003	1.151	**0.053**	1.073	0.999	1.153				
Claudin-2 Low-High	**0.030**	1.719	1.055	2.802	**0.025**	1.767	1.073	2.910	**0.038**	**1.751**	**1.033**	**2.969**
Afadin Low-High	**0.015**	1.674	1.103	2.539	0.056	1.518	0.989	2.329	0.120	1.445	0.908	2.299

Abbreviations: HR, Hazards Ratio; CI, confidence interval; ER, estrogen receptor.
Numbers in bold represent statistically significant differences.
*Each marker was added one at the time in the model with clinical parameter. Results of the clinical parameters were those when associated with Claudin-2 Low-High
**Claudin-2 and Afadin markers were added together in the model with clinical parameter. Results of the clinical parameters were those when associated with both markers.
