# Supplementary material for: Afadin cooperates with Claudin-2 to promote breast cancer metastasis
Source: Genes Dev. 2019 Feb 1;33(3-4):180–93. doi: 10.1101/gad.319194.118 (PMC6362814; doi:10.1101/gad.319194.118)
Supplement: Supplemental Material [file supp_gad.319194.118_Supplemental_Table_S1.pdf]

Tabariès_Supplemental_Table_S1

Supplemental Table S1: 5-years breast cancer specific survival (BCSS).

	Univariate				Multivariate*				Multivariate**			
	P	HR	95.0% CI for HR		P	HR	95.0% CI for HR		P	HR	95.0% CI for HR	
			Lower	Upper			Lower	Upper			Lower	Upper
Age (>50 vs<=50)	**0.046**	1.718	1.010	2.921	-	-	-	-				
ER (+ vs -)	**<0.001**	0.296	0.172	0.510	-	-	-	-				
Grade TMA (3 vs 1-2)	**0.021**	1.995	1.110	3.587	-	-	-	-				
Nodule (N+vsN0)	**0.025**	2.028	1.094	3.761	0.230	1.530	0.764	3.066	0.350	1.399	0.692	2.829
Size (>2cm vs <=2cm)	**0.032**	1.813	1.052	3.126	**0.043**	2.007	1.022	3.945	**0.073**	1.930	0.940	3.962
Claudin-2_Continuous	**<0.001**	1.261	1.127	1.411	**0.000**	1.251	1.118	1.401				
Afadin_Continuous	**0.037**	1.101	1.006	1.205	**0.044**	1.100	1.003	1.207				
Claudin-2 Low-High	**0.003**	3.696	1.566	8.725	**0.003**	3.628	1.533	8.585	**0.008**	3.615	1.406	9.297
Afadin Low-High	**0.008**	2.182	1.223	3.893	**0.015**	2.096	1.157	3.798	0.061	1.832	0.972	3.453

Abbreviations: HR, Hazards Ratio; CI, confidence interval; ER, estrogen receptor.
Numbers in bold represent statistically significant differences.
*Each marker was added one at the time in the model with clinical parameter. Results of the clinical parameters were those when associated with Claudin-2 Low-High
**Claudin-2 and Afadin markers were added together in the model with clinical parameter. Results of the clinical parameters were those when associated with both markers.
