# Supplementary material for: Afadin cooperates with Claudin-2 to promote breast cancer metastasis
Source: Genes Dev. 2019 Feb 1;33(3-4):180–93. doi: 10.1101/gad.319194.118 (PMC6362814; doi:10.1101/gad.319194.118)
Supplement: Supplemental Material [file supp_gad.319194.118_Supplemental_Table_S5.pdf]

Supplemental Table S5: Clinical and pathological characteristics of the 206 patients

	Frequency	Percentage
**Breast Cancer Specific Mortality**		
no	47	22.8
yes	159	77.2
**Relapse-Free Survival**		
yes	192	93.2
Missing	14	6.8
**Liver Metastasis Free Survival**		
no	115	55.8
yes	91	44.2
**Lung Metastasis Free Survival**		
no	139	67.5
yes	67	32.5
**Metastatic Site Category**		
Loco-regional	35	17
Bone	37	18
Lung	43	20.9
Liver	91	44.2
**Mstage**		
0	180	87.4
1	25	12.1
Missing	1	0.5
**Nstage**		
N0	67	32.5
N+	121	65.1
Missing	5	2.4
**Tstage**		
<20	83	40.3
>20	122	59.2
Missing	1	0.5
**Histological Grade**		
1/2	79	38.3
3	103	50
Missing	24	11.7
**PR**		
negative	81	39.3
positive	110	53.4
Missing	15	7.3
**ER**		
negative	36	17.5
positive	158	76.7
Missing	12	5.8
**HER2**		
negative	175	91.1
positive	17	8.3
Missing	14	6.8

Abbreviations: ER, estrogen receptor; PR, progesterone receptor
