# Supplementary material for: Afadin cooperates with Claudin-2 to promote breast cancer metastasis
Source: Genes Dev. 2019 Feb 1;33(3-4):180–93. doi: 10.1101/gad.319194.118 (PMC6362814; doi:10.1101/gad.319194.118)
Supplement: Supplemental Material [file supp_gad.319194.118_Supplemental_Figure_S7.pdf]

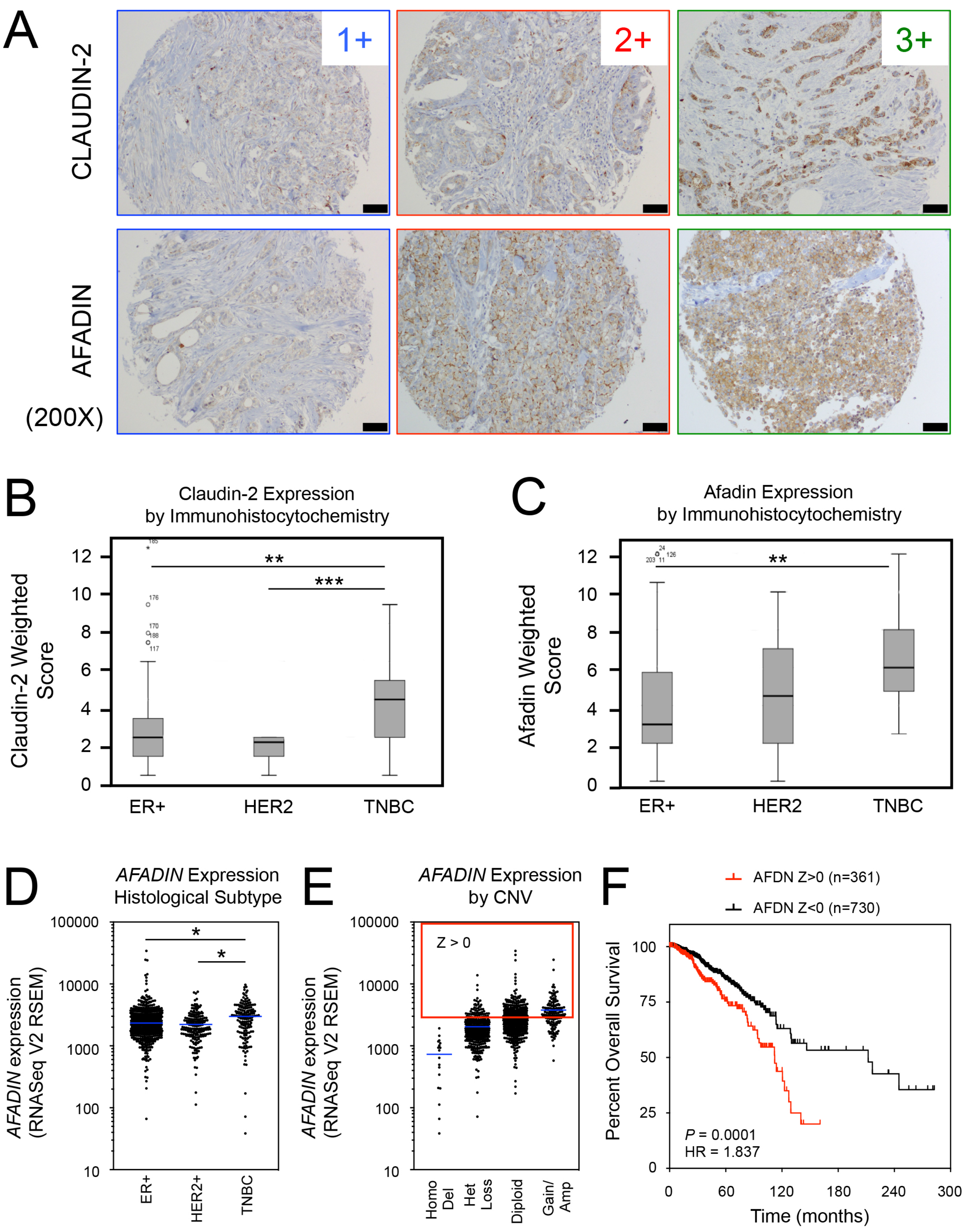
A
1+
2+
3+
CLAUDIN-2
AFADIN
(200X)
B
Claudin-2 Expression
by Immunohistocytochemistry
Claudin-2 Weighted Score
**
***
ER+
HER2
TNBC
C
Afadin Expression
by Immunohistocytochemistry
Afadin Weighted Score
**
ER+
HER2
TNBC
D
AFADIN Expression
Histological Subtype
AFADIN expression (RNASeq V2 RSEM)
ER+
HER2+
TNBC
E
AFADIN Expression
by CNV
Z > 0
Homo Del
Het Loss
Diploid
Gain/ Amp
F
AFDN Z>0 (n=361)
AFDN Z<0 (n=730)
Percent Overall Survival
Time (months)
P = 0.0001
HR = 1.837
