# Supplementary material for: Afadin cooperates with Claudin-2 to promote breast cancer metastasis
Source: Genes Dev. 2019 Feb 1;33(3-4):180–93. doi: 10.1101/gad.319194.118 (PMC6362814; doi:10.1101/gad.319194.118)
Supplement: Supplemental Material [file supp_gad.319194.118_Supplemental_Table_S3.pdf]

Supplemental Table S3: 5-years liver metastasis free survival (LiMFS).

	Univariate				Multivariate*			
	P	HR	95.0% CI for HR		P	HR	95.0% CI for HR	
			Lower	Upper			Lower	Upper
Age (>50 vs<=50)	0.163	1.456	0.859	2.467	-	-	-	-
ER (+ vs -)	0.347	0.708	0.345	1.454	-	-	-	-
Grade TMA (3 vs 1-2)	0.77	1.085	0.628	1.873	-	-	-	-
Nodule (N+vsN0)	**0.019**	2.065	1.126	3.786	0.155	1.708	0.817	3.572
Size (>2cm vs <=2cm)	**0.019**	1.904	1.112	3.26	0.385	1.346	0.689	2.629
Claudin-2_Continuous	0.061	1.146	0.994	1.322	-	-	-	-
Afadin_Continuous	0.128	1.079	0.978	1.191	-	-	-	-
Claudin-2 Low-High	**0.032**	2.346	1.074	5.123	**0.047**	2.214	1.010	4.853
Afadin Low-High	0.148	1.555	0.855	2.83	-	-	-	-

Abbreviations: HR, Hazards Ratio; CI, confidence interval; ER, estrogen receptor.
Numbers in bold represent statistically significant differences.
*Each marker was added one at the time in the model with clinical parameter. Results of the clinical parameters were those when associated with Claudin-2 Low-High
