# Supplementary material for: Afadin cooperates with Claudin-2 to promote breast cancer metastasis
Source: Genes Dev. 2019 Feb 1;33(3-4):180–93. doi: 10.1101/gad.319194.118 (PMC6362814; doi:10.1101/gad.319194.118)
Supplement: Supplemental Material [file supp_gad.319194.118_Supplemental_Table_S6.pdf]

Supplemental Table S6. Primer sequences

name	sequence
Gapdh (sense)	5’-CAAGTATGATGACATCAAGAAGGTGG-3’
Gapdh (antisense)	5’-GGAAGAGTGGGAGTTGCTGTTG-3’
Rims2 (sense)	5’-CTGCTCAGCTAGTGGGACG-3’
Rims2 (antisense)	5’-CCCGGATGATTTCTACCTCCAG-3’
Arhgap21 (sense)	5’- GCTCAGTGAGAATTTGGGAACA-3’
Arhgap21 (antisense)	5’- GTCTTAAAGGCTGTGGCGTCT-3’
